# Inequalities and indoor air pollution: a prospective observational study of particulate matter (PM_2.5_) levels in 309 UK homes from the Born in Bradford cohort study

**DOI:** 10.1186/s12889-025-25182-x

**Published:** 2025-11-10

**Authors:** Rachael W. Cheung, Lia Chatzidiakou, Tiffany C. Yang, Simon P. O’Meara, David R. Shaw, Denisa Genes, Yunqi Shao, Athina Ruangkanit, Thomas Warburton, Ashish Kumar, Sri Hapsari Budisulistiorini, Chantelle Wood, Gordon McFiggans, Nicola Carslaw, Jacqueline F. Hamilton, Rosemary R. C. McEachan

**Affiliations:** 1 Born in Bradford, Bradford Health Research Institute, Bradford Teaching Hospitals NHS Foundation Trust, Bradford, BD9 6RJ UK; 2 Department of Health Sciences, University of York, York, YO10 5DD UK; 3 Yusuf Hamied Department of Chemistry, University of Cambridge, Cambridge, CB2 1EW UK; 4 National Centre for Atmospheric Science, University of Manchester, Manchester, M13 9PL UK; 5 Department of Earth and Environmental Science, University of Manchester, Manchester, M13 9PL UK; 6 Department of Environment and Geography, University of York, York, YO10 5DD UK; 7 National Centre for Atmospheric Science, University of York, York, YO10 5DD UK; 8 School of Psychology, University of Sheffield, Sheffield, S1 4DP UK; 9 Wolfson Atmospheric Chemistry Laboratories, Department of Chemistry, University of York, York, YO10 5DD UK

**Keywords:** Social determinants of health, Particulate matter, PM2.5, Indoor air pollution, Inequalities, Ethnicity, Socioeconomic deprivation, Homes

## Abstract

**Background:**

Particulate matter (PM_2.5_) is associated with substantial morbidity and mortality. Evidence suggests socioeconomic and ethnic minority groups are disproportionately exposed to higher outdoor air pollution, exacerbating existing health inequalities. However, most research focuses on outdoor air pollution, despite people spending most of their time indoors. We compare how indoor PM_2.5_ concentrations vary between households of different socioeconomic status and ethnicity, and test for associations with asthma-related symptoms.

**Methods:**

We recruited 321 households from the multi-ethnic Born in Bradford cohort. Low-cost commercial sensors sampled PM_2.5_ in three rooms over a two-week period. Information on socio-economic status, home and building characteristics, and asthma related symptoms were collected for 309 mothers and 293 children. We calculated metrics for indoor PM_2.5_ concentration (µg/m^3^) to compare with current guideline thresholds and to capture peak events that might be important for health symptoms. We investigated whether PM_2.5_ concentrations varied by key sociodemographic and home characteristics. Logistic regressions examined whether PM_2.5_ metrics predicted asthma-related symptom occurrence for mothers and children, controlling for covariates.

**Results:**

Homes had a mean daily average indoor PM_2.5_ concentration of 20.2 µg/m^3^, exceeded the WHO 24-hour threshold an average of 41% monitored days, and exceeded 100 µg/m^3^ an average of 4% monitored hours. South Asian homes had higher PM_2.5_ concentration than White British or Other ethnicity homes (23.5 µg/m^3^, 17.1 µg/m^3^, and 16.5 µg/m^3^ respectively). Higher PM_2.5_ was observed with higher deprivation levels (most deprived, 24.0 µg/m^3^, least deprived, 12.7 µg/m^3)^. Higher PM_2.5_ levels were seen in rented versus owned homes, smoking versus non-smoking households, terraced and semi-detached versus detached homes, and gas versus electric cooking appliances. We did not find clear associations between asthma-related symptoms and PM_2.5_ metrics.

**Conclusions:**

The high indoor PM_2.5_ levels recorded in homes indicate an urgent need to tackle indoor air pollution as a health risk factor, particularly in deprived and minority ethnic households. Policy action should focus on launching national public awareness campaigns, supporting transition to cleaner cooking and air cleaning technologies, and addressing socioeconomic disparities related to high indoor air pollution.

**Supplementary Information:**

The online version contains supplementary material available at 10.1186/s12889-025-25182-x.

## Background

Air pollution causes significant harm to health [1]. One key component of air pollution is particulate matter (PM). PM is linked to a wide range of poor health outcomes in multiple organ systems [2], and can originate from both natural (e.g. pollen, dust) and anthropogenic (e.g. combustion, cooking) sources. In particular, PM_2.5_ – fine particles of less than 2.5 micrometres in diameter – has been linked to poor cardiovascular, cerebrovascular, and respiratory outcomes. Across 40 European countries in 2020, 275,000 premature deaths were attributed to PM_2.5_ levels [3]. The relative risk of mortality per 10 µg/m^3^ of PM_2.5_ is estimated to be 1.08 (95%CI: 1.06–1.09) [4]. Short-term PM_2.5_ exposure over a two-week period has been associated with an increase in symptoms such as wheeze and cough in children [5] and adults with asthma [6, 7]. Longer-term exposure over a number of years has been associated with reduced lung function and development [8], poorly controlled asthma in both adults [9] and children [10], and an increase in presentation of asthma-related conditions to Accident and Emergency departments [11–14].

To protect people from harmful PM_2.5_ exposures, the World Health Organisation (WHO) set a recommended limit for 24-hour average PM_2.5_ not exceeding 15 µg/m^3^ more than 3–4 days per year, and a limit for annual average PM_2.5_ concentrations of 5 µg/m^3^ [15]. However, much of the underpinning research for this policy has come from outdoor air measurements, with nearly all health studies of air pollution using data from outdoor air quality monitoring networks as metrics of exposure [16].

This work suggests that the burden of exposure to PM_2.5_ levels may not be equally distributed across social determinants of health. Areas of higher socioeconomic deprivation appear to show a general trend of higher outdoor air pollution across North American, Latin America, Asia, and some parts of Europe [17–19]. Ethnicity has been also been associated with outdoor air pollution across different countries, with minority ethnic groups experiencing higher air pollution [20–23]. As minority ethnic groups often experience higher levels of deprivation within residential countries, separation of ethnic and socioeconomic factors is difficult [24]. However, one large-scale study of UK 2021 Census data found all minority ethnic groups experienced higher average PM_2.5_ than White ethnic groups in the same deprivation categories, with Bangladeshi and Pakistani groups experiencing an average of 40% higher outdoor PM_2.5_ emissions locally [25]. Overall, higher exposure to outdoor air pollution may compound long-standing existing health inequalities around ethnicity [26], such as higher risks for respiratory and cardiovascular hospital admissions in Pakistani groups as compared to White British groups [27]. However, compared to research on outdoor PM_2.5,_ the study of indoor PM_2.5_ is less well-established. This is despite calls for a better understanding of exposure to air pollution in indoor environments due to health impacts [28, 29] and despite across industrialised nations, people spend as much as 80–90% of their lives indoors [30], with 56–66% of the day spent inside homes [31]. Limited research currently suggests increased levels of deprivation, indexed by higher occupancy, lower household education, and lower income, have been associated with higher levels of indoor air pollution in the US, Korea, and Europe [32]. However, the lack of further data on indoor PM_2.5_ and general reliance on outdoor metrics, which do not adequately capture people’s exposure during most of their daily lives, limits our understanding of subsequent health impacts.

There is therefore an urgent need to develop effective public health policies or guidance frameworks to reduce exposure to harmful indoor PM_2.5_ concentration levels. To do that, we need to better understand PM_2.5_ levels indoors, their relation with social determinants of health, behavioural factors, and their impact on respiratory health. The current paper highlights key emerging findings from one of the most comprehensive studies of indoor air pollution in homes to date: a cross-sectional, multi-method, indoor air monitoring study within the longitudinal birth cohort study Born in Bradford [33]. This study was part of the wider INGENIOUS project (*understandING the sourcEs*,* traNsformations and fate of IndOor air pollUtants* [34], and involved deploying commercial low-cost air pollution sensors that recorded PM_2.5_ across approximately 300 UK households for two weeks.

## Aims and objectives

This paper aims to describe how PM_2.5_ concentration levels measured inside real homes broadly relate to key social determinants of health and the home and building characteristics collected within the INGENIOUS study. We explored the following research questions:

1. What levels of PM_2.5_ concentration are participants exposed to in the home?
2. How do social determinants of health, such as ethnicity, deprivation, and housing tenure, relate to indoor PM_2.5_ in the home?
3. How do home and building characteristics such as smoking, pet ownership, age of construction, type of property, relate to PM_2.5_ in the home?
4. What are the associations between PM_2.5_ at home and mothers and children’s respiratory symptoms during the data collection period?

## Materials and methods

### Study design

The study design was a prospective observational study, carried out in Bradford, West Yorkshire, United Kingdom with families enrolled in the longitudinal Born in Bradford cohort [33]. Families were recruited and commercial low-cost air quality sensors installed in three rooms (kitchen, living space and child’s bedroom) for two weeks, and information on building characteristics, behaviour and health collected. Full details can be found in [35]. The study was approved by the Bradford Leeds NHS research ethics committee (reference code: 22/YH/0288, 11th January 2023).

### Setting

Bradford is the fifth largest city in the UK, with a population of 560,200, and high ethnic diversity: 32% of the population identify as Asian, the majority of which are South Asian [36]. According to 2021 England and Wales Census data, approximately 57% of households within the Bradford district are classified as deprived in one or more household characteristics (education, employment, health, and housing); higher than the national rate of 52% [37, 38]. Annual PM_2.5_ concentrations outdoors in 2021–2023 ranged from 7.1 to 8.4 µg/m^3^ [39]. Respiratory illness is higher in Bradford district compared to the national average, with 7.4% of the population living with asthma, compared to the national average of 6.5% [40, 41].

### Recruitment and data collection procedure

Families who had taken part in the most recent wave of Born in Bradford data collection (2017–2020) were eligible to take part. Recruitment was stratified by child ethnicity (White British; South Asian; Other), housing tenure (private/mortgaged; rented), and children’s asthmatic status (had active asthma diagnosis recorded in primary care records within 2 years). We aimed for half of the recruited families to include children with asthma. These families were then contacted for inclusion in INGENIOUS. Inclusion criteria were: mother able to give informed consent for themselves, their household, and their children; the household had suitable electricity supplies and space for indoor air quality sensors, and the parent was able to complete questionnaires and diaries. Exclusion criteria were: mother unable to give informed consent for themselves, their household, or their children, and/or unable to communicate in English.

At the initial visit trained researchers completed a building audit and installed the sensors. After two weeks the sensors were removed and participants completed a health and behaviour survey. Participants received a £50 voucher as a token of appreciation for completing the study, and a personalised air quality report at the end of the monitoring period. Further details and an example air quality report can be found in the study protocol [35].

### PM_2.5_ indoor data measurements

The sensors deployed in this study were commercial AirGradient sensor platforms (https://www.airgradient.com/) integrating multiple low-cost sensors (see Supplemental Materials for further information). The sensors captured indoor PM concentration (PM_1_, PM_2.5_, PM_10_ in micrograms per cubic metre, µg/m^3^) temperature (°C), relative humidity (%), carbon dioxide (parts per million), and Total Volatile Organic Compounds (parts per billion by volume) at 1 min resolution, averaged over 5 min. The current paper reports PM_2.5_ levels only. According to international standard BS ISO 16000-37:2019 [42], the deployment research team placed sensors on tables or shelves away from external walls, windows, HVAC inlets and outlets, direct emission sources and direct sunlight, ensured nothing covered the bottom or top of the sensors, and ensured sensor placement did not interfere with occupant activities. Additional information was captured on where sensors were placed relative to windows and the dimensions of the room and can be found in Supplementary Materials (Tables S1 and S2). Measurements were transmitted to a secure server through cellular connection provided by the deployment research team. Remote data capture from sensors was monitored regularly and participants contacted if there was a connectivity issue. Quality assurance procedures and sensor calibration was performed throughout the study, including comparisons with co-located reference instruments (see Supplementary Materials, Figures S2 and S3).

### Building audit and home survey data (Day 1)

**Fig. 1:**
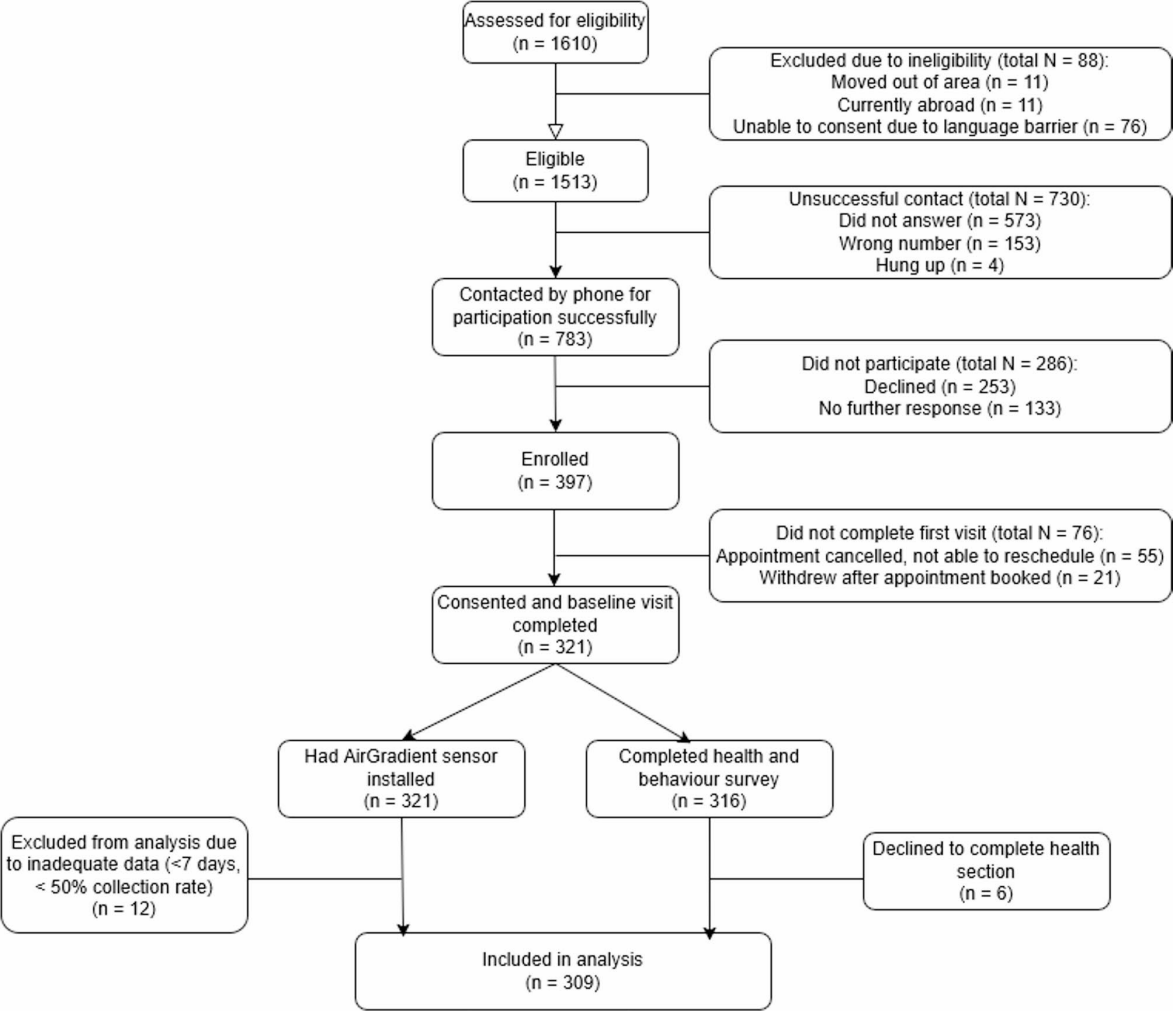
Diagram using CONSORT guidelines for INGENIOUS households in Born in Bradford study

Researchers completed an audit of building characteristics on Day 1 (Fig. 1) at the start of the monitoring period, including if the home was owned by someone in the household or rented (including social housing and private lets), the type of property (flat/apartment, terraced home, semi-detached home, detached home/bungalow), when the home was built (pre-1914, between 1914 and 1964, between 1965 and 1990, after 1991), and heating and cooking appliance type (electric or gas). At the same visit, researchers also asked participants questions on home and behaviour characteristics, including whether anyone in the home smoked cigarettes, e-cigarettes, cigar, or pipes inside or outside (smoking or non-smoking household), if the house had pets (has any pets, or no pets), and when people were usually at home (09:00–14:59; 15:00–17:59; 18:00–22:59; 23–08:59). For the latter, participants could tick multiple options; to provide an estimate of overall self-reported home occupancy, we assigned each block of time 25% and summed the overall time per household that participants were at home (e.g. if a participant only ticked 09:00–14:59, this would be 25%; if a participant ticked all four options, this would be 100%).

### Health and behaviour surveys data (Day 14)

Health surveys included modified questions from the *International Study of Asthma and Allergies in Childhood* (ISAAC; [43] asking mothers to report asthma-related respiratory symptoms for their Born in Bradford child, and the *Global Asthma Network* (GAN [44] surveys to self-report their own asthma-related respiratory symptoms within the two-week period when the sensors were deployed. As both the ISAAC and GAN were originally designed to score symptoms over 12 months, we focussed on symptom occurrence during the two-week period, rather than total scores, with a primary interest in child asthma symptoms. For the ISAAC, we scored occurrence of any respiratory symptom (wheeze, cough, use of asthma medication, and wheeze limiting exercise) over the two-week period as ’1’, and non-occurrence as ’0’. For the GAN, we scored the occurrence of wheeze in adults (occurrence ‘1’, non-occurrence ‘0’).

### Other measures

Child ethnicity was extracted from Born in Bradford records. Child asthma status was taken from primary healthcare records for all children with an active asthma diagnosis within the last two years. We also collected information on Indices of Multiple Deprivation 2019 (IMD-2019 [45], for participants using their address data from primary care records during the recruitment process. IMD-2019 is a geographical measure of relative deprivation by living area used by the UK Government, comprising seven domains (income, employment, health deprivation and disability, education and skills training, crime, barriers to housing and services, living environment), and is split into national deciles across England. However, as Bradford has a larger percentage of highly deprived areas than other English cities, a national scale for deprivation does not capture variation within Bradford. Therefore, the national IMD-2019 raw scores were categorised into quintiles within Bradford, where the 1 st quintile was most deprived, and the 5th was the least.

### Statistical analyses

All data handling, analysis, and visualisation was done in R (v4.4.1 [46], using R Studio (v.4.4) with *base* R, *tidyverse* [47], and *wesanderson* [48] R packages. For all data analyses, we used a complete cases analysis, as our intention was to describe the data as it was collected. Where data were missing this is indicated in Results tables alongside proportion, except for sensor data, which is indicated in Results main text. For sensor data, rooms in homes were retained for further analysis if they fulfilled the following 3 criteria: [1] they had at least 7 valid days in the 14-day period (≥ 50% collection rate); [2] a day was considered valid if there were at least 12 valid hours collected (≥ 50% collection rate); [3] an hour was considered valid if there were at least 6 observations of the 12 maximum (≥ 50% collection rate). Please see [34] for further details.

For sensor data that fitted the inclusion criteria, we first calculated the average indoor PM_2.5_ concentration for each home per day (by adding all 5-minute observations together in a day, and dividing this by the number of observations per day, where one day is 24-hours) at both the home and room level. We used the daily average indoor PM_2.5_ concentration at the home level to calculate the mean daily (24-hour) average indoor PM_2.5_ for the full period of data collection – producing one metric per home. We also calculated the percentage of monitored days (24-hour periods) where the mean daily average indoor PM_2.5_ exceeded the WHO 24-hour threshold of 15 µg/m^3^, by summing the number of days where the average daily indoor PM2.5 was over 15 µg/m^3^ and dividing this by the total number of days collected, then multiplying this by 100. The WHO 24-hour threshold metric was chosen to provide information on homes recording days above policy-derived thresholds.

We also calculated the average hourly indoor PM_2.5_ concentration for each home (by adding all 5 min observations together in an hour, and dividing this by the number of observations per hour) at both the home and room level, and used this to calculate the total percentage of hours collected where mean hourly average indoor PM_2.5_ is over 100 µg/m^3^. The hourly threshold metric was calculated by summing the number of hours over 100 µg/m^3^, dividing this by the total number of hours collected, then multiplying this by 100 and chosen to provide information on time spent at a persistently high threshold.

We report descriptive statistics and data trends for each metric by home in the main text; descriptive statistic and data trends for each sensor location (kitchen, living room, child’s bedroom) are in Supplemental Materials. For general home and building characteristics, we report the mean PM_2.5_ and standard deviation as additional descriptive information. To test for group-based differences in PM_2.5_ metrics between ethnicity, housing tenure, and deprivation specified as variables of a priori interest in the protocol [35], we conducted two-sample unpaired Wilcoxon tests (housing tenure) and Kruskal-Wallis tests with pairwise Wilcoxon tests using false discovery rate [49] corrections for *p*-values (ethnicity, deprivation), as the data were not normally distributed. We also report general descriptive information about how deprivation indices and ethnicity, housing tenure, and building characteristics co-occur. Finally, we conducted logistic regressions separately for mothers and children, examining whether the occurrence of respiratory symptoms was predicted by the mean daily average indoor PM_2.5_, the mean percentage of monitored hours over the WHO threshold, and the mean percentage of monitored hours over 100 µg/m^3^, controlling for age of participant, deprivation, ethnicity, prior asthma diagnosis, and smoking status of household, with an additional co-variate of child sex for child outcomes. We used treatment coding, where coefficients are calculated relative to a reference level (ethnicity, reference = ‘White’; asthma, reference = ‘none’; smoking, reference = ‘non-smoker’; IMD-2019, reference = ‘most deprived’; housing tenure, reference = ‘own’; season of sensor deployment, reference = ‘Winter’).

## Results

The study recruited 321 households in total (Fig. 1) between 9th March 2023 and 19th April 2024. Household recruitment was distributed between seasons with 31% of households participating in Spring (20th March to 20th June), 25% in Summer (21st June to 22nd September), 22% in Autumn (23rd September to 21 st December), and 22% in Winter (22nd December to 19th March). Sociodemographic characteristics of households recruited were similar to recruitment targets in the protocol [35]. A total of 49% South Asian (target: 45%), 41% White British (target: 45%), and 10% Other (target: 10%) households were collected. A total of 46% of recruited households had a record of a Born in Bradford child having an active asthma diagnosis (target: 50%), and 76% reported living in private (either mortgaged, or living with person with mortgage) homes (target: 70%), with the remaining 24% living in rented homes (private or social housing; target: 30%).

Occupant ethnicity in the 1 st – 3rd deprivation quintiles was skewed towards South Asian ethnicity, as was Other ethnicity, whereas for White ethnicity, it was skewed towards the 3rd – 5th quintiles (Supplementary Materials, Figure S4A). The most deprived quintiles also appeared to have higher proportions of rented homes than the least, although home ownership was skewed towards the most deprived homes as well (Figure S4B). Homes in the 1 st – 3rd quintiles tended to be terraced and semi-detached homes (Figure S4D); however, there was little pattern identified between the age of the home and deprivation quintiles (Figure S4C) and notably, a third of this data on age of the home was missing.

### Total PM_2.5_ home exposure metrics

After applying the inclusion criteria, we retained measurements from 309 homes out of the 321 households that completed all questionnaire surveys. In total, over 3.5 million observations were retained for analysis corresponding to ~ 13,850 home-room-days and ~ 300,000 home-room-hours. Per home, this was an average of 13.6 days (SD = 1.6) and 925.2 h (SD = 124.2). Participants reported being at home on average 76% of the day (SD = 29%; minimum 25%, maximum 100%). Table 1 shows the data collected and total PM_2.5_ metrics per home over the monitoring period (collapsed across all rooms). Table S3 in Supplemental Materials shows the same metrics but with a breakdown per room.

**Table 1:** Mean (*M*) and standard deviation (*SD*) of PM_2.5_ concentration metrics by recruitment strata across the INGENIOUS data collection period

Variable	Levels (*n*, % of sensor sample)	Daily average indoor PM_2.5_ (µg/m^3^)		Monitored days, daily average indoor PM_2.5_ exposure > 15 µg/m^3^ (%)		Monitored hours, hourly average indoor PM_2.5_ exposure > 100 µg/m^3^ (%)	
		M	SD	M	SD	M	SD
Overall	*N* = 309, 100%	20.2	25.7	41	32	4	7
Ethnicity	South Asian (*150*, 49%)	23.5	26.7	51	31	5	7
	Other (*32*, 10%)	16.6	21.0	33	29	3	7
	White (*127*, 41%)	17.2	25.1	31	30	3	8
Housing tenure	Rent (*73*, 24%)	23.9	26.5	51	32	5	8
	Own (*236*, 76%)	19.0	25.3	38	32	4	7
IMD-2019 BFD quintile	1 st quintile (most deprived, *65*, 21%)	23.8	24.4	52	33	5	6
	2nd quintile (*84*, 27%)	22.7	24.6	48	32	5	8
	3rd quintile (*82*, 27%)	20.7	32.3	37	32	4	10
	4th quintile (*49*, 16%)	14.3	18.9	28	28	2	4
	5th quintile (least deprived, *23*, 7%)	12.8	15.2	26	27	2	3
	Missing (*6*, 2%)	16.3	11.9	41	18	2	2
Child asthma status	Asthma (*144*, 47%)	18.5	21.4	39	32	4	6
	No asthma (*164*, 53%)	21.7	28.9	43	33	5	9
	Missing (*1*, < 1%)	-	-	-	-	-	-
Smoking household	Smoker (*117*, 38%)	27.0	33.6	51	36	6	11
	Non-smoker (*188*, 61%)	16.0	18.1	35	28	3	4
	Missing (*4*, 1%)	17.0	17.1	39	23	2	2
Pets	Has pets (*135*, 44%)	20.3	26.6	40	33	4	8
	No pets (*174*, 56%)	20.1	24.9	42	31	4	7
Age of building	Pre-1914 (*56*, 18%)	17.2	21.4	38	28	3	5
	Between 1914–1964 (*67*, 22%)	23.1	32.7	42	33	5	10
	Between 1965–1990 (*33*, 11%)	25.3	35.2	46	34	6	13
	After 1991 (*50*, 16%)	17.7	21.3	35	32	3	5
	Missing (*103*, 33%)	19.5	19.9	44	33	4	5
Type of building	Detached/bungalow (*48*, 16%)	15.1	18.0	32	26	2	4
	Flat (*5*, 2%)	18.7	14.5	52	37	3	4
	Semi-detached (*150*, 49%)	21.0	27.9	42	32	4	8
	Terraced (*106*, 34%)	21.6	25.6	44	34	4	8
Cooking appliance	Electric (*107*, 35%)	17.8	23.9	35	31	3	7
	Gas (*198*, 64%)	21.7	26.7	45	33	5	8
	Missing (*4*, 1%)	9.0	8.4	18	15	1	1
Sensor deployment	Winter (*67*, 22%)	25.1	23.5	58	32	6	6
	Spring (*93*, 30%)	21.0	31.6	37	31	4	10
	Summer (*77*, 25%)	14.2	18.7	29	28	2	5
	Autumn (*72*, 23%)	20.8	24.1	44	32	4	7

*IMD-2019 BFD* Index of Multiple Deprivation 2019, Bradford District

The mean daily average indoor PM_2.5_ concentration was 20.2 µg/m^3^ (SD = 25.7 µg/m^3^). On average, homes spent 41% (SD = 32%) of monitored days over the recommended WHO 24-hour threshold for indoor PM_2.5_ levels, ranging from 0% *(n* = 37 homes) to 100% (*n* = 20 homes), meaning some homes spent no days over the threshold, and some spent all monitored days above the recommended 24-hour limit. On average, homes had 4% (SD = 7%) of monitored hours during the 2-week period over 100 µg/m^3^ PM_2.5,_ ranging from 0% (*n* = 21 homes) to 68% (*n* = 1 home), again indicating high between-home variations in indoor PM_2.5_ levels.

**Fig. 2:**
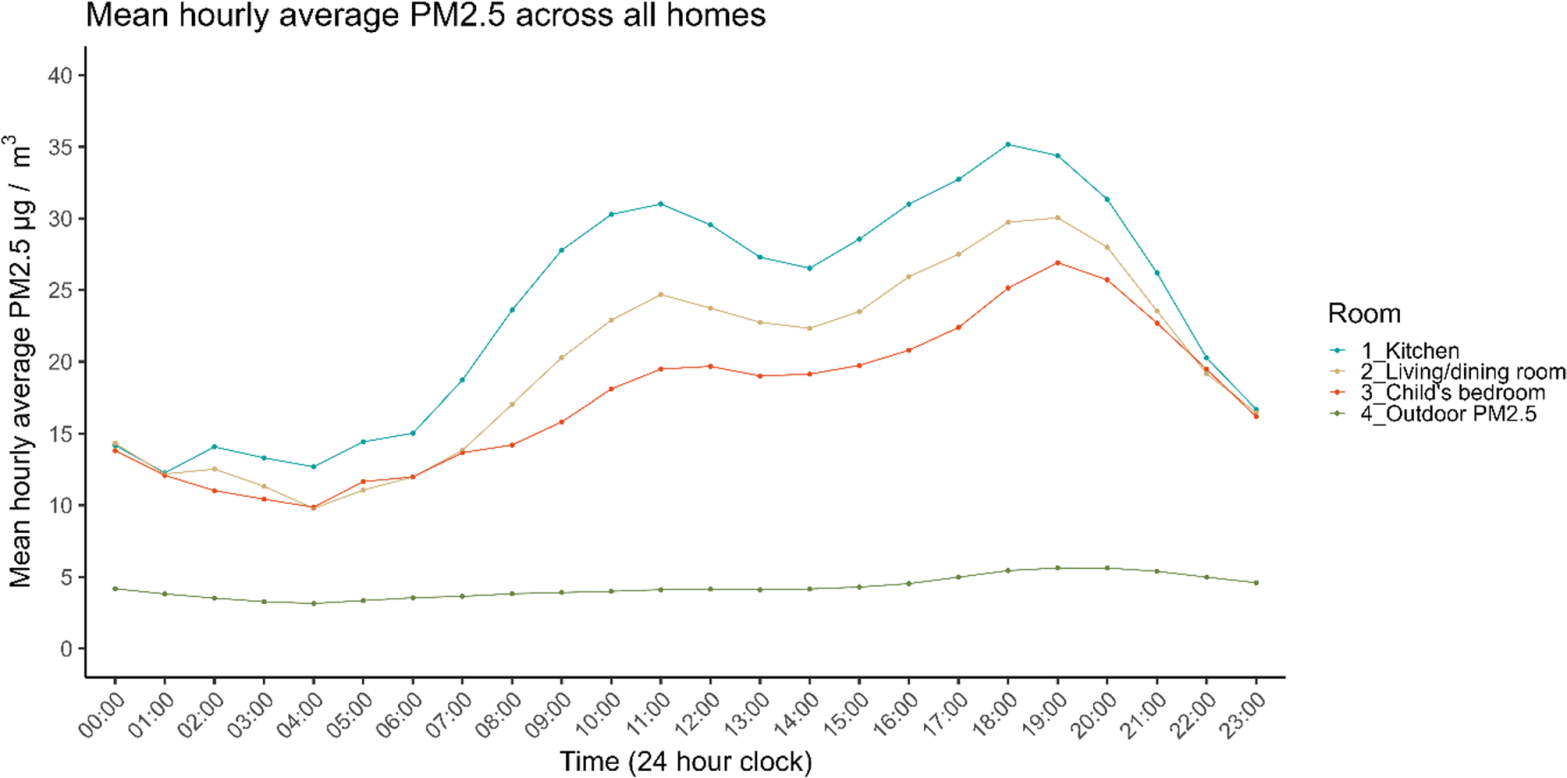
Mean hourly average indoor PM_2.5_ µg/m^3^ concentration measured by AirGradient sensors across all homes per room. Mean hourly average outdoor PM_2.5_ levels provided by City of Bradford Metropolitan Council and Automatic Urban Rural Network from the Department for Environment, Food, & Rural Affairs (please see [35] and Supplemental Materials for further details)

The lowest daily average indoor PM_2.5_ concentrations were found in Summer (M = 14.2 µg/m^3^, SD 18.7 = µg/m^3^), and highest in Winter (M = 25.1 µg/m^3^, SD = 23.5 µg/m^3^). Consistent with these general patterns, of the 37 homes that spent 0% of days over the WHO 24-hour threshold, most were collected in Spring (*n* = 11 homes) and Summer (*n* = 18), with the remainder in Autum and Winter (both *n* = 4); of the 20 homes that spent 100% of days over the threshold, they were evenly distributed between Spring (*n* = 7), Autumn (*n* = 6), and Winter (*n* = 6), with one home in Summer. Additional plots of PM_2.5_ by month of data collection are in Supplemental Materials (Figure S5, Table S4) and show a similar seasonal pattern. Mean hourly average indoor PM_2.5_ concentrations across homes were highest during the day and lowest overnight (Fig. 2). Kitchens had the highest mean daily average indoor PM_2.5_ concentration of 23.5 µg/m^3^, followed by living/dining rooms at 19.7 µg/m^3^, and children’s bedrooms at 17.3 µg/m^3^ (Fig. 2, Table S3).

### PM_2.5_ by home and building characteristics

Means and standard deviation alongside group sample sizes can be found in Table 1; further breakdown by room can be found in Table S3 in Supplemental Materials. Homes with smokers had higher daily average indoor PM_2.5_ concentration than non-smokers (*M* indoor PM_2.5_ = 27.0 µg/m^3^ versus *M* indoor PM_2.5_ = 16.0 µg/m^3^, respectively). Homes with smokers exceeded the WHO 24-hour threshold 51% of monitored days on average and had a mean of 6% of monitored hours over high thresholds of 100 µg/m^3^, whereas non-smoking homes had a mean of 35% of monitored days and 3% of monitored hours exceeding thresholds. Indoor PM_2.5_ concentrations between homes who had pets (*M* daily average indoor PM_2.5_ = 20.3 µg/m^3^) were similar to those without pets (*M* daily average indoor PM_2.5_ = 20.1 µg/m^3^).

The age of the building was missing for 33% of the sample. Compared to homes built before 1914, between 1914 and 1964, and after 1991, homes built between 1965 and 1990 appeared to have the highest daily average indoor PM_2.5_ levels (*M* = 25.3 µg/m^3^; see Table 1). On average, they also exceeded the WHO 24-hour threshold of 46% of monitored days, and exceeded the 100 µg/m^3^ hourly threshold concentration 6% of monitored hours. Terraced homes had the highest PM_2.5_ concentration, with a mean daily average indoor PM_2.5_ of 21.6 µg/m^3^, a mean 44% of monitored days over the WHO 24-hour threshold, and a mean 5% of monitored hours over the 100 µg/m^3^ threshold. These values were similar to semi-detached homes (see Table 1). Although flats had the highest percentage of monitored days over the WHO 24-hour threshold – 52% of monitored days – flats comprised only 2% of the total sample size.

### PM2.5 by social determinants of health

**Fig. 3:**
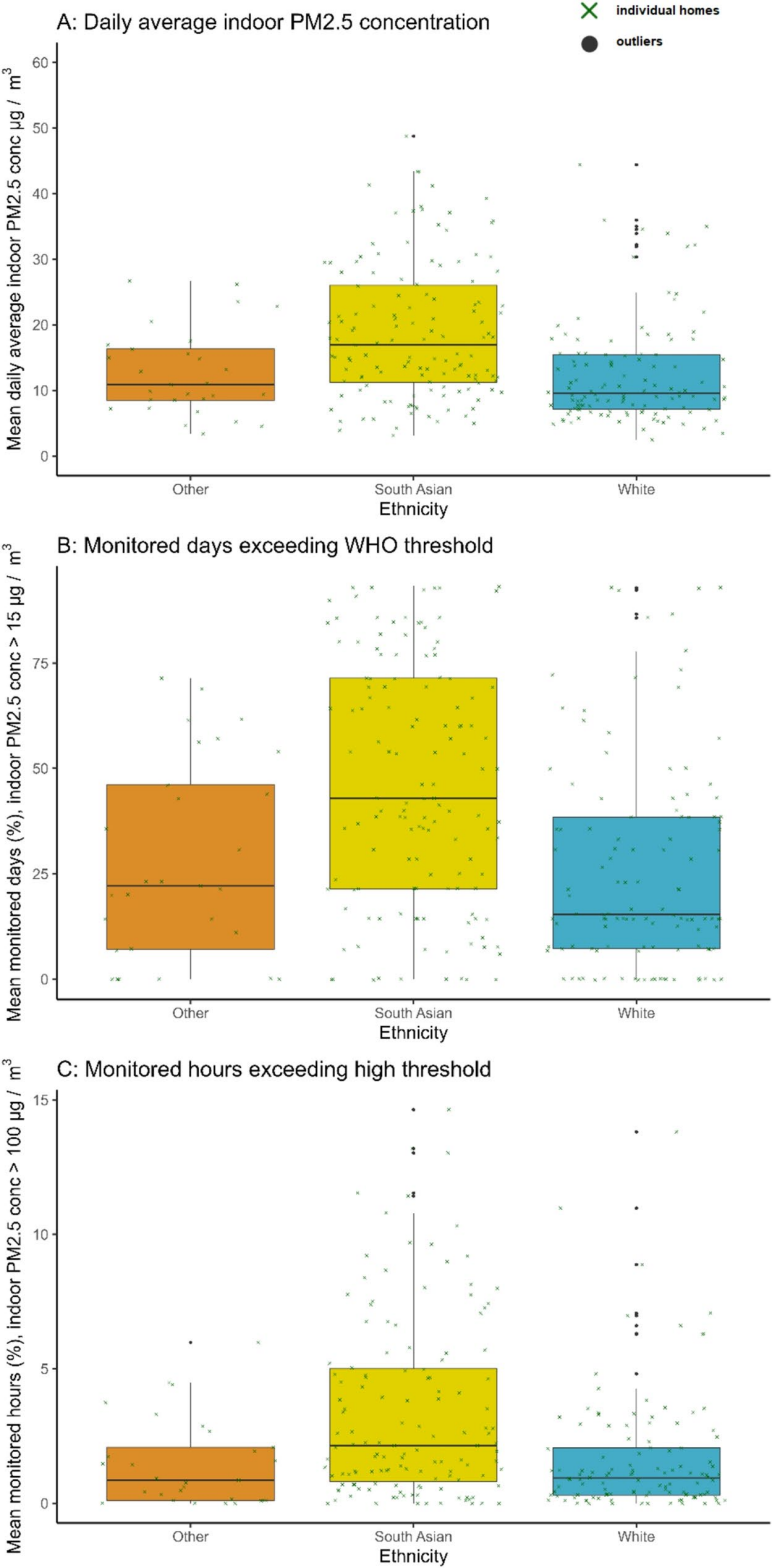
Box-and-whisker plots showing the median and interquartile range by ethnicity for indoor PM_2.5_ metrics for the 95th percentile of the data per home: (**A**) mean daily average indoor PM_2.5_ concentration (µg/m^3^); (**B**) mean percentage of monitored hours where hourly average indoor PM_2.5_ exceeds 100 µg/m^3^; (**C**) mean percentage of monitored days where daily average indoor PM_2.5_ exceeds 15 µg/m^3^ (WHO 24-hour threshold). All values are shown at the home level, amalgamating data from three sensors (kitchen, living/dining room, child’s bedroom)

**Fig. 4:**
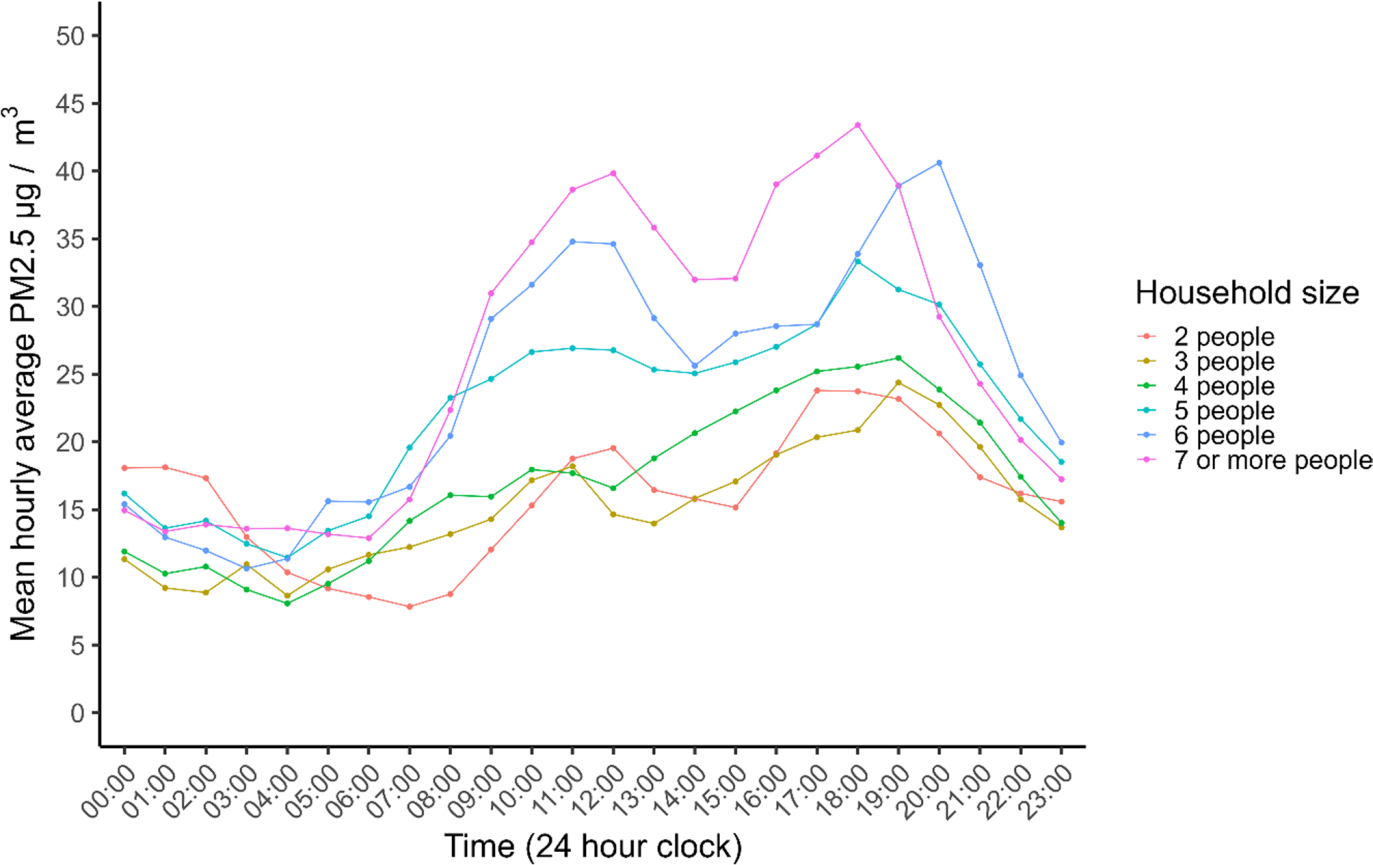
Mean hourly average indoor PM_2.5_ concentration by household size for 95th percentile of the AirGradient data (*N* = 282 homes)

**Fig. 5:**
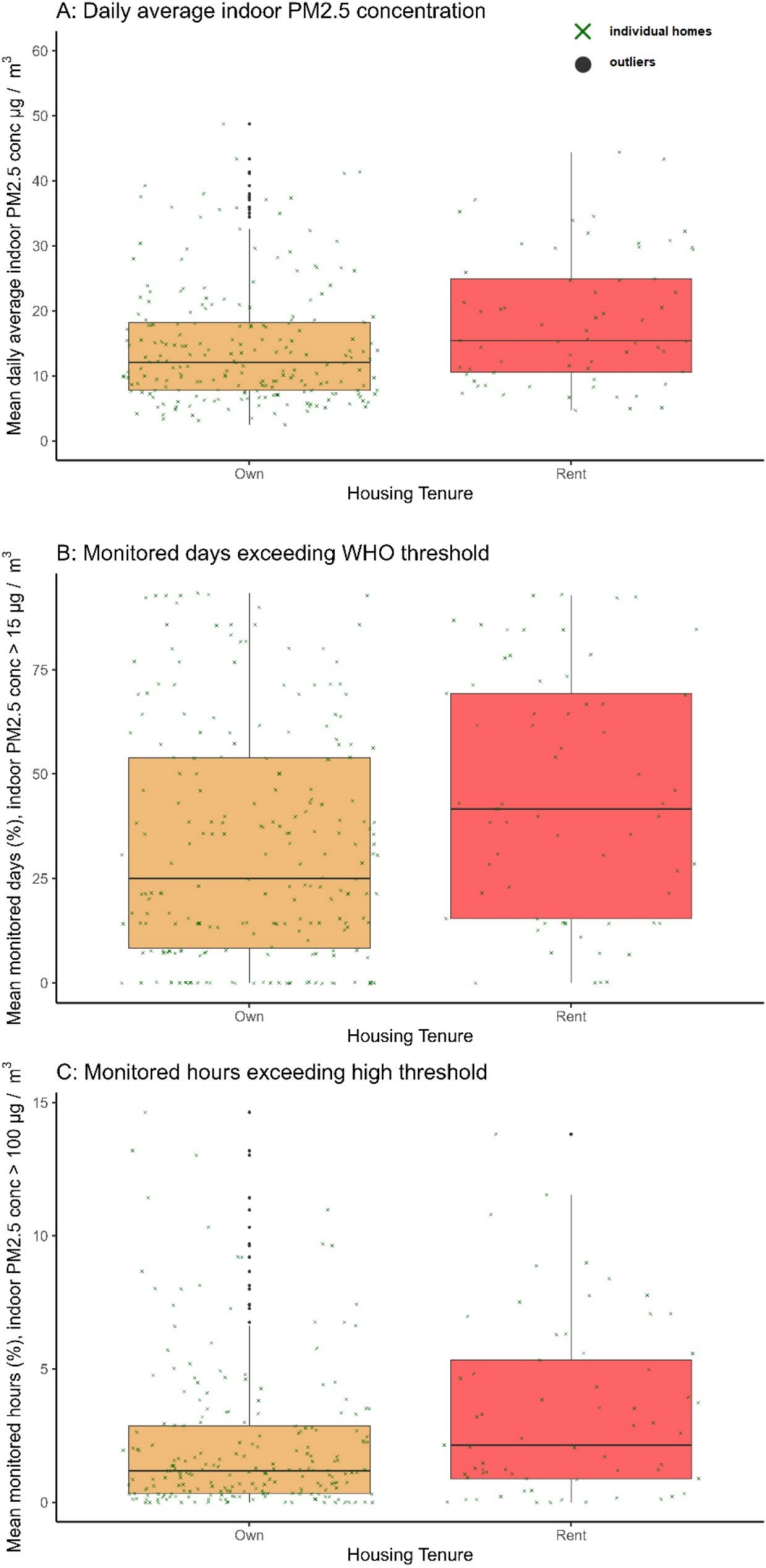
Box-and-whisker plots showing median and interquartile range by housing tenure for indoor PM_2.5_ metrics for the 95th percentile of the data per home: (**A**) mean daily average indoor PM_2.5_ concentration (µg/m^3^); (**B**) mean percentage of monitored hours where hourly average indoor PM_2.5_ exceeds 100 µg/m^3^; (**C**) mean percentage of monitored days where daily average indoor PM_2.5_ exceeds 15 µg/m^3^ (WHO 24-hour threshold). All values are shown at the home level, amalgamating data from three sensors (kitchen, living/dining room, child’s bedroom)

**Fig. 6:**
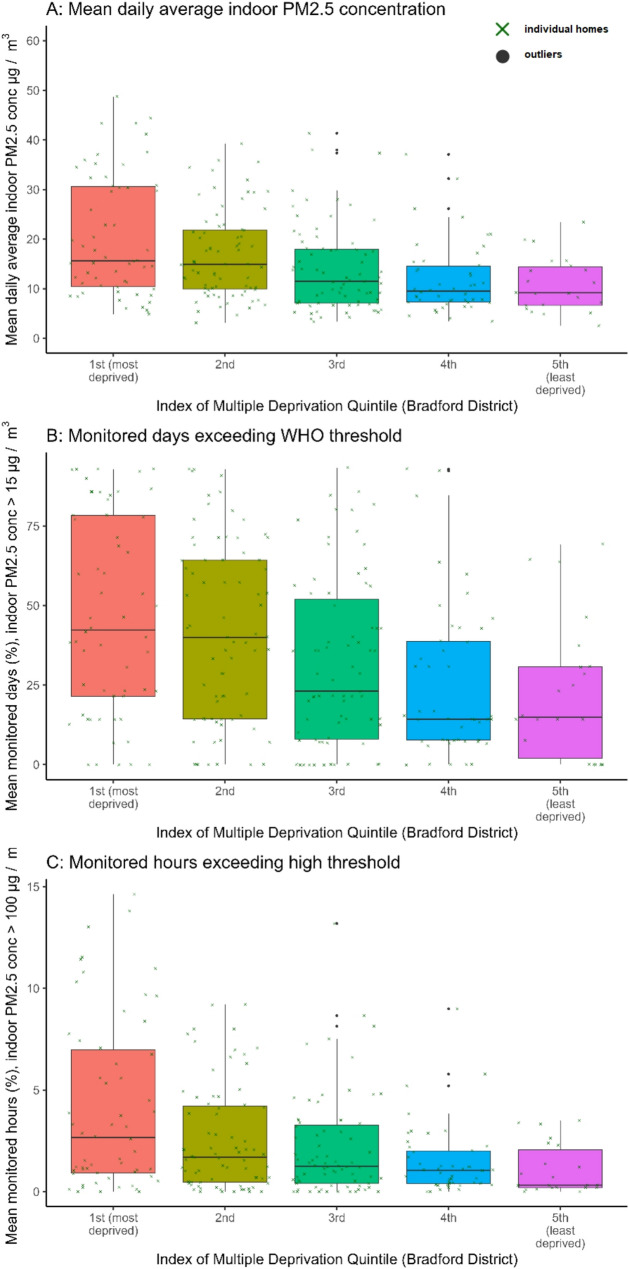
Box-and-whisker plots showing median and interquartile range by Index of Multiple Deprivation 2019 Bradford district quintiles for indoor PM_2.5_ metrics for the 95th percentile of the data per home: (**A**) mean daily average indoor PM_2.5_ concentration (µg/m^3^); (**B**) mean percentage of monitored hours where hourly average indoor PM_2.5_ exceeds 100 µg/m^3^; (**C**) mean percentage of monitored days where daily average indoor PM_2.5_ exceeds 15 µg/m^3^ (WHO 24-hour threshold). All values are shown at the home level, amalgamating data from three sensors (kitchen, living/dining room, child’s bedroom)

Descriptive statistics for ethnicity, housing tenure, and deprivation quintiles can be found in Tables 1 and 3; Figs. 3 and 5, and 6. Figure 3 shows the distribution of mean daily average indoor PM_2.5_ by ethnicity. South Asian homes had higher mean levels of daily average indoor PM_2.5_ (23.4 µg/m^3)^ than Other (16.6 µg/m^3)^ and White British homes (17.2 µg/m^3^; *Kruskal-Wallis H* [2] = 30.95, *p* <.001). Over the monitoring period South Asian homes spent a mean of 51% of days above the WHO 24-hour threshold, as compared to Other and White British homes, which exceeded the WHO 24-hour threshold a mean of 31% and 33% of monitored days respectively (*H* [2] = 30.12, *p* <.001). Finally, South Asian homes also spent more hours at average indoor PM_2.5_ thresholds >100 µg/m^3^ (5%) as compared to Other (3%) and White British (3%) homes (*H* [2] = 25.26, *p* <.001) during the sensor deployment period. Across all three metrics, pairwise comparisons using Wilcoxon rank sum tests identified South Asian homes had significantly higher indoor PM_2.5_ across all metrics as compared to Other and White British homes, whereas White British and Other homes did not differ significantly (Tables 1 and 2). Due to these results, we also examined the number of people in a household by ethnic group post-hoc; South Asian homes had a mean and median of 5 people (minimum = 2, maximum = 12), whereas Other and White British both had a mean and median of 4 people (minimum 2, maximum 8). We also plotted mean hourly average indoor PM_2.5_ concentration by household size (number of people) and found a general trend of larger household size and higher indoor PM_2.5_ levels (Fig. 4). Examining the household size by ethnic group for these data (Table 2) demonstrated South Asian homes tended to have higher numbers of people within their household than White or Other homes.

**Table 2:** Household size by total sample and by ethnic group (*n* = 307; two homes were missing household size)

Household size	*N*, total sample	*N*, South Asian	*N*, White British	*N*, Other
2 people	19	5	10	4
3 people	50	13	30	6
4 people	81	20	54	7
5 people	75	45	23	7
6 people	50	41	4	5
7 or more	32	25	5	2

**Table 3:** Results of pairwise comparisons using Wilcoxon rank sum tests for mean PM_2.5_ concentration metrics by key social determinants of health

	South Asian			Other								
Ethnicity	***Daily average*** ***PM***_***2.5***_	***% days > 15*** ***µg/m***^***3***^	***% hours > 100*** ***µg/m***^***3***^	***Daily average*** ***PM***_***2.5***_	***% days > 15*** ***µg/m***^***3***^	***% hours > 100*** ***µg/m***^***3***^	-		- -	-		-
White	< 0.001	< 0.001	< 0.001	0.546	0.648	0.594	-		- -	-		-
Other	0.004	0.005	0.002	-	-	-	-		-	-		-
Housing tenure	**Rented**											
	***Daily average*** ***PM***_***2.5***_	***% days > 15*** ***µg/m***^***3***^	***% hours > 100*** ***µg/m***^***3***^	-		-	-		-	-		-
Own	0.003	0.003	0.006	-		-	-		-	-		-
IMD-2019 BFD	** 1 st quintile (most deprived)**			**2nd quintile**			**3rd quintile**			**4th quintile**		
	***Daily average*** ***PM***_***2.5***_	***% days > 15*** ***µg/m***^***3***^	***% hours > 100*** ***µg/m***^***3***^	***Daily average*** ***PM***_***2.5***_	***% days > 15*** ***µg/m***^***3***^	***% hours > 100*** ***µg/m***^***3***^	***Daily average*** ***PM***_***2.5***_	***% days > 15*** ***µg/m***^***3***^	***% hours > 100*** ***µg/m***^***3***^	***Daily average*** ***PM***_***2.5***_	***% days > 15*** ***µg/m***^***3***^	***% hours > 100*** ***µg/m***^***3***^
2nd	0.321	0.412	0.193	-	-	-	-	-	-	-	-	-
3rd	0.007	0.011	0.031	0.031	0.056	0.244	-	-	-	-	-	-
4th	< 0.001	0.002	0.003	0.001	0.004	0.044	0.213	0.192	0.193	-	-	-
5th (least deprived)	0.003	0.005	0.003	0.005	0.011	0.031	0.209	0.192	0.079	0.765	0.772	0.200

*IMD-2019 BFD* Index of Multiple Deprivation 2019, Bradford District

Figure 5; Table 1, and Table 3 show that rental homes including private lets and social housing had higher mean daily average indoor PM_2.5_ concentrations (23.9 µg/m^3^) than owned homes (19.0 µg/m^3^; *Wilcoxon rank sum test [W]* = 6578, *p* =.003). Rented homes had a mean of 51% of monitored days over the WHO 24-hour threshold, as compared to owned homes, which spent a mean of 38% monitored days over this threshold (*W* = 6619.5, *p* =.003). Rented homes also had slightly higher hourly average indoor PM_2.5_ above 100 µg/m^3^ than owned homes (means; 5% versus 4% respectively; *W* = 6774, *p* =.006).

Figure 6; Tables 1 and 3 show a trend of higher indoor PM_2.5_ concentrations and increased time spent over PM_2.5_ thresholds with increasing deprivation (mean daily average indoor PM_2.5_, *H* [4] = 27.89, *p* <.001; WHO 24-hour threshold, *H* [4] = 24.34, *p* <.001; hours >100 µg/m^3^, *H* [4] = 20.24, *p <*.001). Across all three metrics, homes from the most deprived quintiles had significantly higher PM_2.5_ than the least deprived (Tables 1 and 3). For example, compared to homes from the least deprived quintile, homes from most deprived homes had a mean daily average indoor PM_2.5_ concentration of 23.8 µg/m^3^ (compared to 12.8 µg/m^3^), 53% of monitored days over the WHO 24-hour threshold (compared to 25%), and 5% of monitored hours over 100 µg/m^3^ (compared to 2%).

### Asthma-related respiratory health symptoms

A total of 293 children (mean age [SD] = 14.6 [1.1] years, range = 12.2–16.7 years, 53% male) and 307 mothers (mean age [SD] = 44.1 [5.6] years, range = 30.5–59.2 years) had data for respiratory health analyses. A total of 47% of children had asthma, with 28% of children reported to have at least one respiratory symptom during the data collection period of two weeks. Table 4 shows a breakdown of all asthma-related respiratory symptoms in children at the end of the two-week period. Overall, 25% of mothers reported a previous diagnosis of asthma for themselves and 11% reported wheeze over the last two weeks.

**Table 4:** Asthma-related respiratory symptoms reported in children (*n* = 293) over the two-week data collection period

Symptom	*n*	% of sample
Wheeze	27	9
Cough	37	13
Use of asthma medication	52	18
Wheeze after exercise	30	10
Shortness of breath impacting speech	3	1
Kept awake by wheeze	13	4

### Asthmatic symptoms in children

The unadjusted and adjusted logistic regression models (Table 5) did not identify a significant association between occurrence of any asthma-related symptom in the two week period and mean daily average indoor PM_2.5_ concentration (Model 1, *adjusted OR* = 1.01, *95%CI* [0.99, 1.02]), mean percentage of monitored days with daily average indoor PM_2.5_ concentration > 15 µg/m^3^ (Model 2, *adjusted OR* = 1.01, *95%CI* [1.00, 1.02]), or mean percentage of monitored hours with hourly average indoor PM_2.5_ concentration > 100 µg/m^3^ (Model 3, *OR* = 1.03, *95%CI* [0.99, 1.07]).

**Table 5:** Odds ratios, 95% confidence intervals, and p-values for logistic regression models predicting occurrence of asthma-related symptoms in the two week data collection period by PM_2.5_ concentration

PM_2.5_ exposure metric	Model type	Outcome: asthma-related respiratory symptoms in children			Outcome: wheeze in mothers		
		OR	95% CI	*p*-value	OR	95% CI	*p*-value
Model 1: daily average indoor PM_2.5_ exposure (µg/m^3^)	Unadjusted	1.01	0.99, 1.02	0.351	1.01	0.99, 1.02	0.440
	Adjusted *	1.01	1.00, 1.02	0.131	1.00	0.98, 1.02	0.968
Model 2: Monitored days, daily average indoor PM_2.5_ exposure > 15 µg/m^3^ (%)	Unadjusted	1.00	1.00, 1.01	0.264	1.00	0.99, 1.01	0.935
	Adjusted *	1.01	1.00, 1.02	0.344	1.00	0.98, 1.01	0.478
Model 3: Monitored hours, hourly average indoor PM_2.5_ exposure > 100 µg/m^3^ (%)	Unadjusted	1.02	0.98, 1.05	0.339	1.02	0.98, 1.06	0.255
	Adjusted *	1.03	0.99, 1.07	0.113	1.02	0.96, 1.06	0.500

*adjusted for covariates: ethnicity, asthma status, age, sex (children only), household smoking status, Index of Multiple Deprivation 2019 Bradford district quintile, household tenure, season of sensor deployment

### Asthmatic symptoms in adults

The unadjusted and adjusted logistic regression models (Table 5) did not identify a significant association between occurrence of wheeze in the two week period and mean daily average indoor PM_2.5_ concentration (Model 1, *adjusted OR* = 1.01, *95%CI* [0.99, 1.02]), mean percentage of monitored days with daily average indoor PM_2.5_ concentration > 15 µg/m^3^ (Model 2, *adjusted OR* = 1.00, *95%CI* [0.99, 1.01]), or mean percentage of monitored hours with hourly average indoor PM_2.5_ concentration > 100 µg/m^3^ (Model 3, *adjusted OR* = 1.02, *95%CI* [0.96, 1.06]).

## Discussion

In a sample of over 300 homes in Bradford UK monitored over approximately two weeks, we found that homes had daily average indoor PM_2.5_ concentrations above recommended thresholds (15 µg/m^3^) 41% of monitored days and extreme high hourly levels (> 100 µg/m^3^) 4% of monitored hours. As participants reported that approximately 76% of their time was spent in the home, there is potential for household members to be exposed to harmful levels of PM_2.5_. These findings highlight the need for urgent further research around understanding and reducing indoor PM exposure in homes. There were inequalities in exposure, with higher indoor PM_2.5_ concentrations and exceedances above thresholds observed in South Asian homes, homes located in more deprived areas, and rental homes. We did not find any clear relation between indoor PM_2.5_ exposure and asthma-related symptoms in children or risk of wheeze in adults over the 2-week study period.

High levels of indoor PM_2.5_ found in this study extend and confirm previous research that shows estimated weighted mean indoor PM_2.5_ across studies to be 16.8 µg/m3 in North America and 23.1 µg/m^3^ in Western Europe – with all regions globally except Oceania over the WHO level of 15 µg/m^3^ [50]. Overall, the mean daily average indoor PM_2.5_ concentration in our study of 20.2 µg/m^3^ was within the range reported in other studies. Outdoor air quality monitoring across Bradford by the city council has generally reported lower outdoor PM_2.5_ levels – the annual mean outdoor PM_2.5_ in 2023 ranged from 7.1 to 8.4 µg/m^3^ [51]. Although indoor PM_2.5_ also includes particles derived from outdoors, our data combined with broader results from the INGENIOUS study [34] suggested occupant activities dominated indoor PM_2.5_, which requires further investigation. Descriptive analyses of PM_2.5_ by home and building characteristics indicated houses constructed between 1965 and 1990, those with gas cooking appliances, and those with smokers had higher PM_2.5_ than other categories. This is consistent with work that finds higher PM in homes with gas cooking appliances [52] and with smoking [53]. Possible mechanisms underlying PM differences by building type relate to natural ventilation efficiency, such as cross-sided ventilation in detached homes as compared to terraced or semi-detached homes, and building regulation changes following the 1973 oil crisis that led to increased air tightness [54], but the relation of indoor air quality with UK building age and associated mechanisms remain unclear [55].

Across ethnicity, tenure, deprivation, home, and building characteristics, the standard deviation and interquartile ranges were notably broad for all PM_2.5_ metrics, indicating high variation within groups; true differences between groups may not be as stark when this individual variation is accounted for. For example, the mean daily average PM_2.5_ of 20.2 µg/m^3^ across all homes was exceeded by standard deviations of 25.7 µg/m^3^. However, some clear patterns were still apparent. South Asian homes had the highest PM_2.5_ levels across all metrics. In particular, they exceeded the WHO 24-hour threshold for PM an average of half of the two-week data collection period, as compared to a third of the two-week data collection period by White British and Other homes. Alternatively, higher PM_2.5_ might reflect different household sizes, where a larger household size results in higher PM_2.5_ concentration, as everyday human activity both generates and resuspends PM_2.5_ [52, 56]. In our sample, South Asian families had a mean and median of 5 people in the household, with a maximum of 12, whereas Other and White British families had a mean and median of 4 people, with a maximum of 8 people. Although we did not have fine-grained occupancy data, we did identify higher indoor PM_2.5_ levels appeared to co-occur with larger household size, consistent with other literature [57] – suggesting higher occupancy relates to more PM_2.5_ generation and possibly resuspension activities. In particular, when observing patterns by occupancy, homes with 5 or more people showed higher PM_2.5_ throughout the day as compared to those with 4 or less people. Alternatively, patterns may reflect different cooking practices between South Asian, Other, and White British homes. Higher PM appears to co-occur with pan-frying compared to boiling and when cooking lentil-based dishes for a long time [58]. Research has found different emission signatures for volatile organic components and different PM concentrations depending on both cooking methods (frying, boiling, etc.) as well as the types of spices and herbs used within controlled simulated kitchen laboratory experiments [59–61]. Future studies will benefit from understanding multiple occupant behaviour in more detail, potentially also by using methodologies such as computational model simulations to better understand individual impacts of cooking and cleaning events [34] and canister samples of indoor air to identify specific composition and sources of PM_2.5_ [34, 62, 63]. Overall, additional future research that investigates cooking and occupant behaviour with social determinants of health in much larger samples are necessary to better understand how these combined factors affect indoor PM_2.5_ concentrations within real homes.

Rented homes also had higher PM_2.5_, spending on average 51% monitored days over the WHO 24-hour threshold as compared to 38% of monitored days in private homes. One report of low-income households from the Institute for Fiscal Studies of the English Housing Survey found rental homes were of poorer quality across electrical safety, sanitation, repair, thermal comfort, and modern facilities, than owner-occupied homes [64]. Some suggest these factors may also link to inadequate ventilation and higher housing density with adjoining buildings [65]. Continued work in INGENIOUS will examine ventilation in the sample relative to building characteristics. Our results also indicated higher PM_2.5_ levels co-occurred with higher deprivation. Compared to the least deprived IMD quintile, the most deprived quintile had 11.3 µg/m^3^ higher mean 24-hour average PM_2.5_ concentration levels (24.0 µg/m^3^ versus 12.7 µg/m^3^) and had twice the mean total number of days spent over the WHO 24-hour threshold (52% vs. 26%). This general trend is consistent with the wider literature that finds higher indoor air pollution correlates with higher deprivation (for a review, see [32]. Possible contributing factors are higher smoking rates in homes with higher deprivation [66]. Additional data from occupancy surveys indicates those receiving government financial support also spend more time at home and have higher overcrowding rates [65], increasing the period of time in which PM_2.5_ can be generated and resuspended indoors. Of note is that South Asian and other homes in the sample tended to belong to more deprived quintiles than White homes, and the distribution of rented homes was higher in more deprived as compared to less deprived homes, although home ownership was also prominent in deprived quintiles. Further research that is designed and powered to detect the differential contributions of these factors is thus warranted.

There was little difference between children with asthma and those without in terms of PM_2.5_ concentration and threshold metrics, with no clear link between PM_2.5_ measured during data collection and asthma-related symptoms in children or mothers. As higher exposure to air pollution may compound long-standing existing health inequalities around ethnicity [26], such as higher risks for respiratory and cardiovascular hospital admissions in Pakistani groups as compared to White British groups [27], the effects of indoor PM_2.5_ home exposure may be difficult to isolate. Whilst the underlying mechanisms between PM_2.5_ and health are not fully understood, PM_2.5_ deposits throughout the respiratory tract likely cause damage via a longer process of oxidative stress, airway inflammation, airway hypersensitivity, and airway remodelling [67]. It is thus likely future studies need longer monitoring periods to capture cumulative effects. Existing literature has thus generally identified either larger ’signals’ of poor respiratory health, e.g. asthma-related emergency visits/admissions [68] or has tracked participant symptoms or asthma diagnosis incidence over a longer period of time than two weeks [69]. In addition, studies generally use outdoor PM_2.5_ rather than indoor measurements, where outdoor PM_2.5_ correlates with other traffic-related pollutants that also cause respiratory symptoms. Where associations between self-reported symptoms and PM_2.5_ concentration have been recorded within two weeks, this has been in small pilot samples of asthmatic populations with pre-existing respiratory hypersensitivity, and using personal exposure sensors that can monitor participants throughout the day [6, 7]. Future studies that examine the indoor environment over a longer monitoring period that can better identify poor respiratory health or use personal exposure sensors are thus warranted.

### Strengths and limitations

Our study has multiple strengths, including the largest indoor air quality sample of over 300 homes in a multi-ethnic city in the UK to date and the first to detail both indoor PM_2.5_ concentrations and inequalities related to these. Our ability to sample three rooms per property over a 2-week monitoring period has provided one of the largest and most intensive indoor air quality datasets in UK homes. We were able to measure exposure at home level and link to individual level observations of ethnicity and socioeconomic status – something that has not been done in previous research. We have a multi-ethnic sample, including groups that are seldom heard in research, with rich information on households and people to allow further exploration of inequalities in exposure. Finally, our findings that homes were frequently exposed to levels above WHO recommendations are highly policy relevant, responding to calls for public health metrics on indoor air pollution from the Chief Medical Officer in the UK [29].

Our study also has some limitations that warrant caution around over generalising results. Social determinants of health such as ethnicity, deprivation indices, and housing tenure likely overlap, and our study does not examine the differential contributions of each of these to PM_2.5_, nor interactions between them. Rather, our results report vital insight into firstly, the high concentration levels of PM_2.5_ measured within homes, and secondly, how patterns of indoor PM_2.5_ differ by key social determinants that require urgent further investigation in larger samples over a longer period of time.

Homes in our study were also sampled in different seasons due to practical reasons of data collection. While the contribution of outdoor-generated PM indoors may vary between seasons driven by factors such as meteorology and active ventilation behaviours (such as occupants opening windows), overall, outdoor variation was relatively small compared to the contribution of indoor sources that dominated measured indoor PM concentrations (see Figure S5, Supplemental Materials, and [34]. Future work will focus on drivers affecting seasonal variation of indoor/outdoor ratios alongside scale separation and source identification as outlined in [34].

Our occupancy data only captured an estimate of when people were at home and household size; it did not account for the number of people in the home in real-time over the two week period, meaning we cannot account for differences in occupancy on a day-to-day basis but only capture overall trends and patterns. We also did not account for holidays or special circumstances that might account for variance in indoor quality or occupant behaviour. A potential solution for future research would be using real-time personal PM_2.5_ monitors combined with geolocation devices and interactive diary smartphone applications to more accurately ascertain how occupancy affects indoor PM_2.5_.

Finally, although large in terms of the amount of indoor air sensor data, our sample of homes is relatively small in public health terms and we had only a short period of time of two weeks to explore health-related impact. The short time frame also limits our understanding of how the two-week data collection period relates to longer-term PM_2.5_ concentration levels; however, future work will aim to understand how representative a two-week period of monitoring is compared with annual deployment in a subsample of participants.

Overall, generalisation of our results beyond homes sampled requires further study in larger national and international samples and must be done with caution, particularly given the high variation between homes. However, our findings provide an important starting point for indoor air quality, particularly in underserved communities, and largely align with broader literature. For example, a recent study in the US found indoor PM_2.5_ concentrations were inversely associated with median household income and positively associated with increased percentage of ethnic minority groups [70]. Of note is that their analysis was carried out based on the local community characteristics in the geographical region of the measurement (Zip Code Tabulation Area), rather than those of individual households; one strength of our study is that we had individual ethnicity as well as neighbourhood deprivation. Similar sized studies also find comparable results to our study regarding indoor PM_2.5_, smoking, and cooking practices [71–73]. Furthermore, assessing pollutant concentration in homes is time-consuming and expensive, and it may be unfeasible at a very large scale. Where possible, using common methods for pollutant concentration, exposure, and health measurement across the research field, will help to build up a larger body of literature and offer a variety of options for meta-analysing smaller studies from diverse areas. Further research, including advanced analytical methods to differentiate indoor and outdoor sources, can better delineate potential PM_2.5_ sources, and thus impacts on health to better inform policy.

## Conclusions

This paper investigated the impact of social determinants, building characteristics and behavioural patterns on indoor PM using one of the largest and most intensive indoor air quality datasets in UK homes. Our study found that homes were routinely exposed to high indoor PM_2.5_ concentrations exceeding the WHO recommendations, with evidence that ethnic minority groups and those living in more deprived areas experienced higher concentrations. To tackle indoor air pollution, possible actions may involve different actors at different levels. This ranges from policy and regulation that can reduce indoor air pollution levels, such as reducing emissions from building materials, fabrics, and furniture, to the development and evaluation of interventions for changing occupant behaviours that impact on indoor air pollution. Possible interventions include supporting replacement of cooking appliances in favour of electric rather than gas, and improving ventilation behaviours during high emitting activities (opening windows, using exhaust fans), and improving ventilation infrastructure in old and new homes [74]. More broadly, public awareness campaigns that offer simple, culturally relevant messaging in multiple language and formats and that partner with community services are likely necessary. Further research is necessary to determine the long-term health and health service use impact of being routinely exposed to such concentrations. As participants spent close to three-quarters of their day within their homes, this means potentially high exposure for families to harmful levels of PM_2.5_. Overall, the results of this study call for further urgent investigation to better delineate indoor sources of household air pollution and their effects on health, particularly for the most vulnerable groups.

## Supplementary Information

Supplementary Material 1

## Data Availability

The datasets used and/or analysed during the current study are available from Born in Bradford on reasonable request. Applications can be made via an expression of interest form available on the study website (https://borninbradford.nhs.uk/our-data/how-to-access-data/) which also includes details on data access fees.

## Abbreviations

GAN: Global Asthma Network
IMD-2019: Indices of Multiple Deprivation 2019
ISAAC: International Study of Asthma and Allergies in Childhood
NHS: National Health Service
PM: Particulate matter
UK: United Kingdom
WHO: World Health Organisation
