# Supplementary material for: Inequalities and indoor air pollution: a prospective observational study of particulate matter (PM2.5) levels in 309 UK homes from the Born in Bradford cohort study
Source: BMC Public Health. 2025 Nov 10;25:3876. doi: 10.1186/s12889-025-25182-x (PMC12604391; doi:10.1186/s12889-025-25182-x)
Supplement: Supplementary file 1 — Supplementary Material 1 [file 12889_2025_25182_MOESM1_ESM.docx]

**Supplemental Material for manuscript ‘Inequalities and indoor air pollution: A prospective observational study of particulate matter (PM_2.5_) levels in 309 UK homes from the Born in Bradford cohort study’**

**The AirGradient sensors**

The AirGradient integrates a series of sensors (Figure S1): a SenseAir S8 using non-dispersive infrared technology to measure CO_2_ concentration (parts per million by volume (ppm)); a Plantower PMS5003 sensor with laser scattering technology to measure three size fractions of PM concentration (PM_1_, PM_2.5_, PM_10_ in micrograms per cubic metre (𝜇g/m^3^)); and a Sensirion SHT3x/4x sensor to measure temperature and relative humidity. The platforms also integrate a Sensirion SGP41 sensor to measure total volatile organic compounds (TVOCs) concentrations (parts per billion by volume (ppb)); however, due to the complexity of quantifying TVOCs with metal oxide sensors, these measurements were only used as a qualitative indicator of indoor emissions.

Further detail: protocol (doi: 10.1136/bmjopen-2023-081099); overview paper (doi: 10.1039/D4EM00634H).

**Figure S1. The AirGradient Sensor**

**
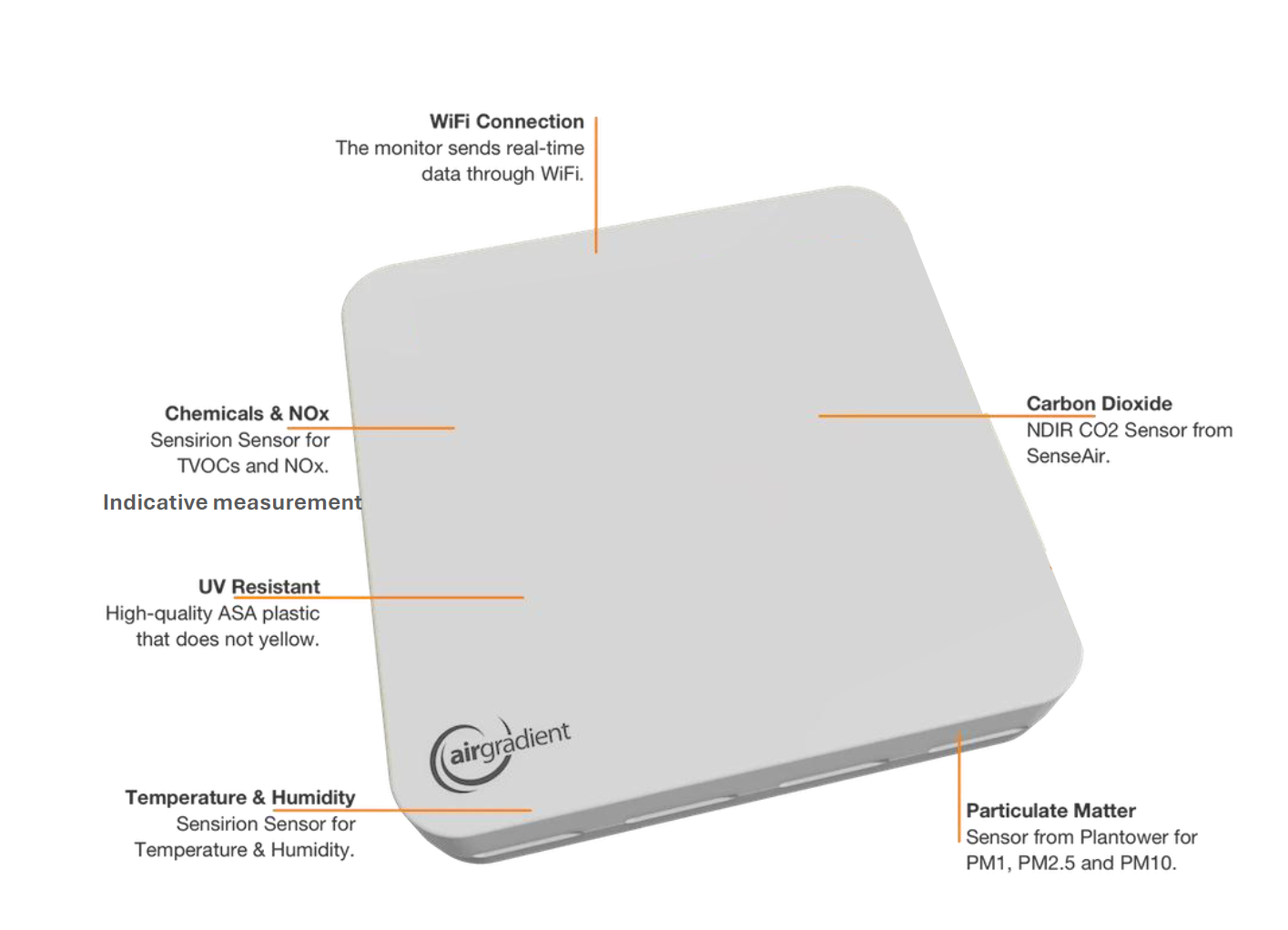
**

**The performance of the PMS5003 sensors integrated in the AirGradient platform**

There were 17 sets of 3, i.e. 51 sensors, deployed. During deployment, daily checks between indoor and outdoor levels were performed (Figure S2, Figure S3 below). Regular indoor co-locations in the office were performed between deployments to assess reproducibility and any sensitivity/offset changes, Overall, sensors were robust with good performance. Our prior work has previously shown that the error of the low-cost sensors is significantly smaller than the error introduced when using inappropriate exposure metrics (i.e. measurements from outdoor reference stations) especially in homes where strong indoor sources operate, significantly elevating indoor concentrations (see Chatzidiakou et al., 2019, doi: 10.5194/amt-12-4643-2019).

**Long-term drift:** During the INGENIOUS deployment, we had 6 “drift” sensors co-located in an urban background site next to a reference FIDAS PALAS 200 S (Figures S2 and S3 below). The performance and reproducibility of the sensors (R2 >0.90, see Figure S3) remained stable over time with very high correlation with the reference instrument (R2 >0.9 for all sensors) and linearity slope close to 1. We did not see an increase in the RMSE (root mean square error) during the deployment.

**Figure S2: Time series of low cost-sensors collocated next to a reference instrument in an urban background setting over 18 months during the INGENIOUS deployment.**


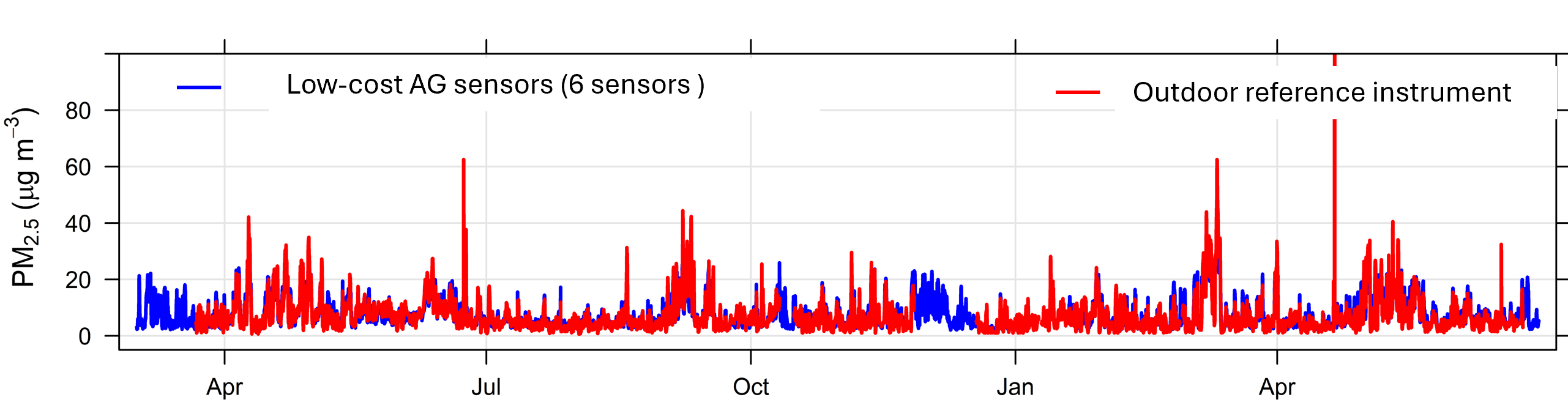


**Figure S3. Colocation deployment of 6 LCS PM sensors outdoors next to a reference instrument. In all cases, the reproducibility between sensors and agreement with reference instrument was high. Linearity was close to 1 and R2>0.90**


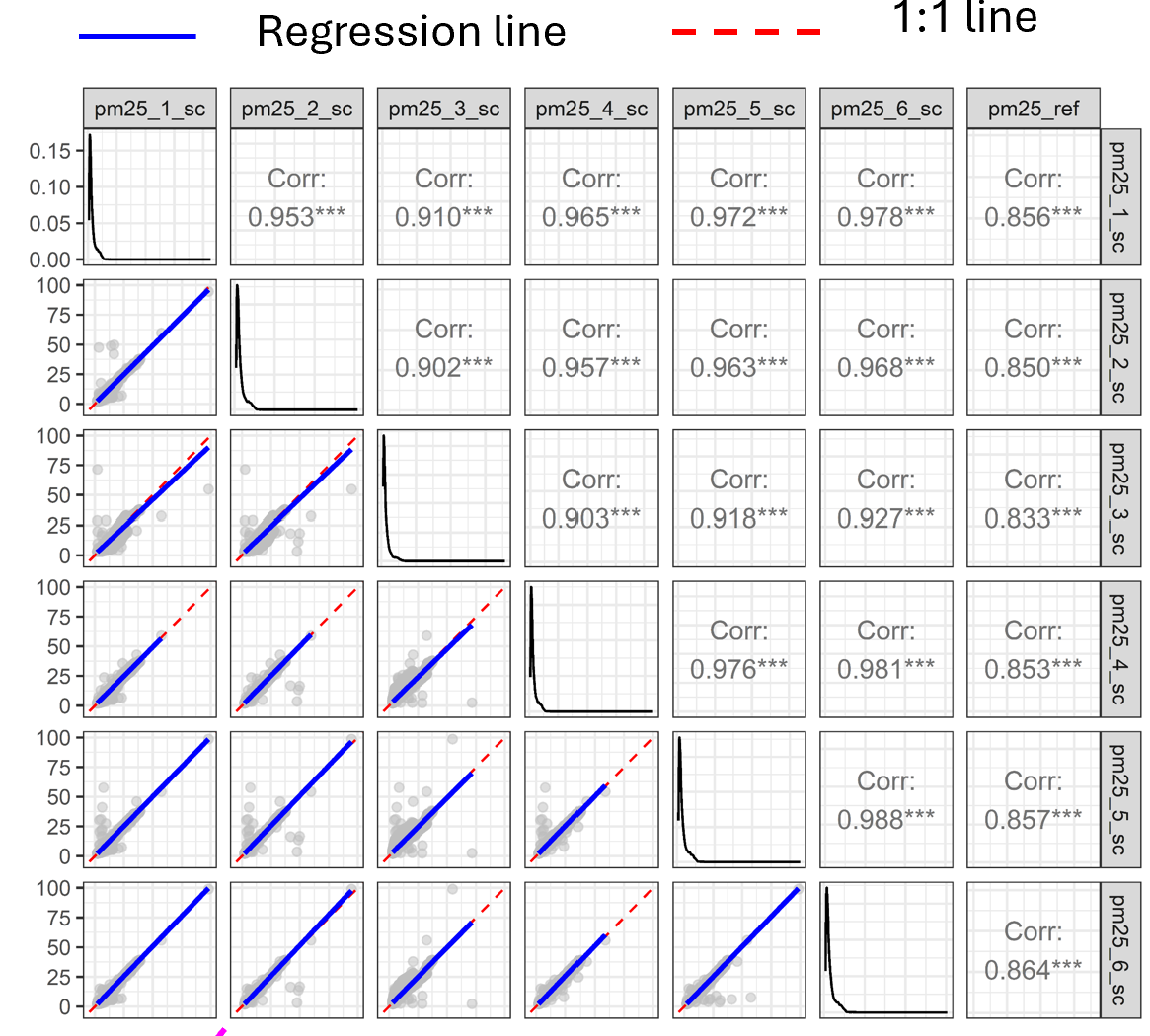


**Figure S4. Co-occurrence of deprivation with key characteristics in sample (N = 321 recruited households).**

**
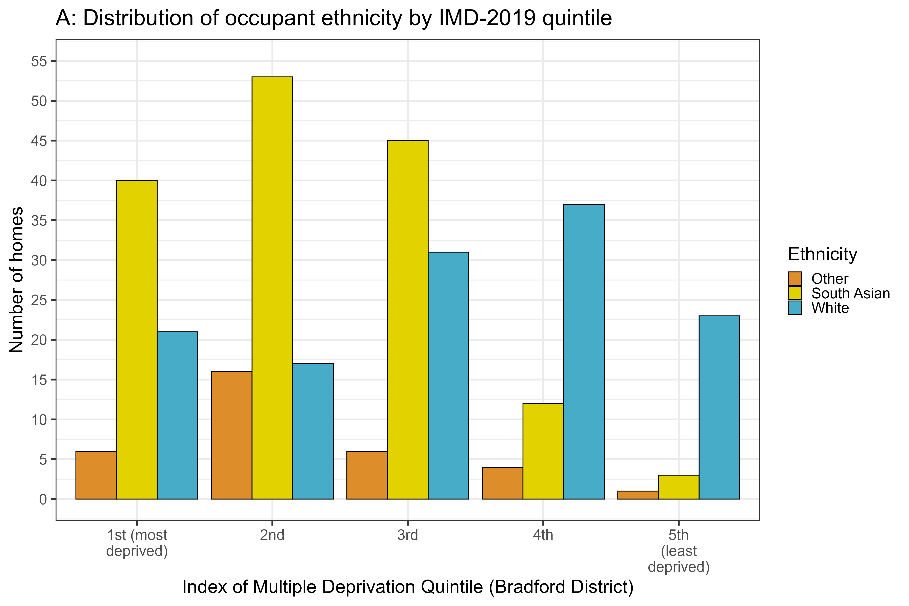
**

**
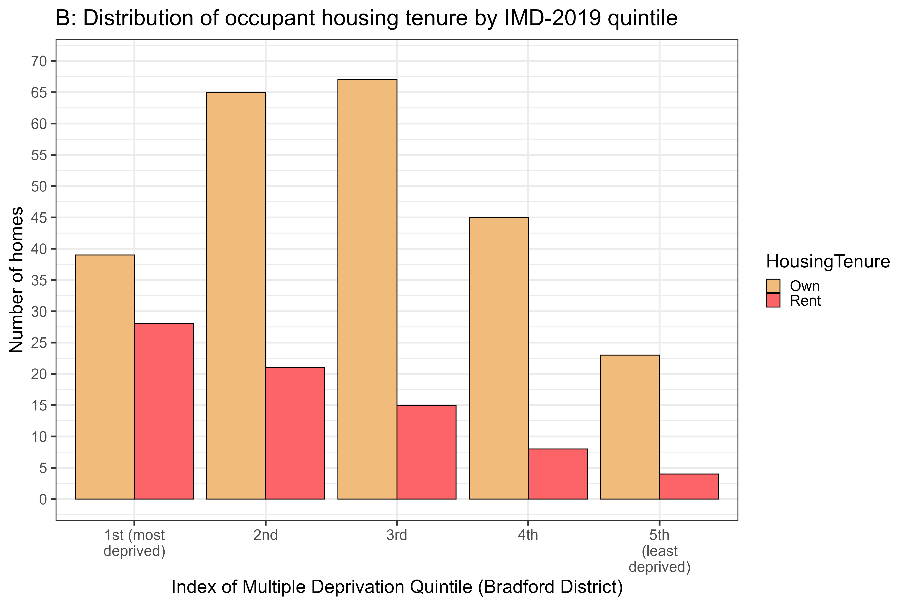
**

**
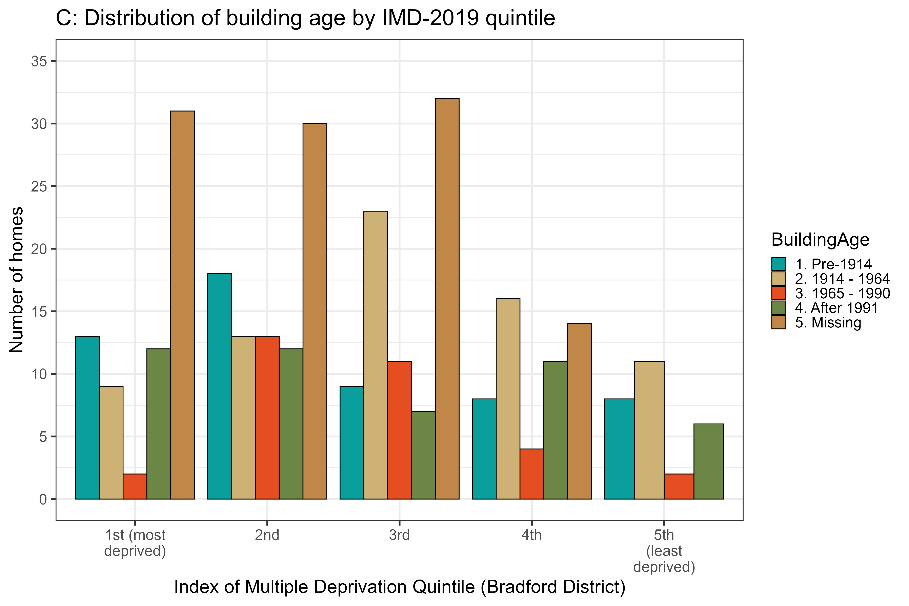
**

**
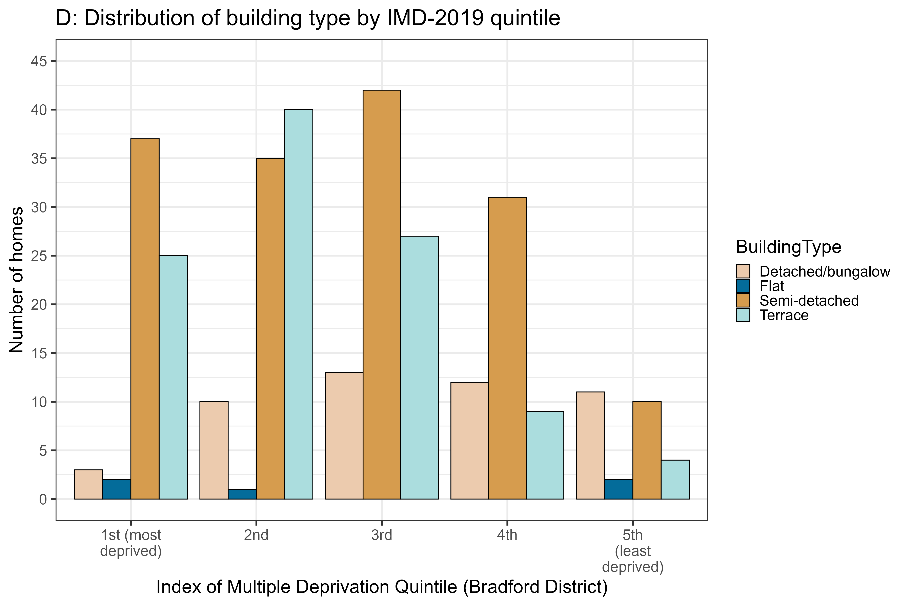
**

**Figure S5. Box-and-whisker plots showing median (black line) and interquartile range (box) of mean indoor PM_2.5_ concentration values from AirGradient sensors within homes and outdoor PM_2.5_ concentration values from Bradford Council and AURN (DEFRA)* by month for the 95^th^ percentile of the data.**

**
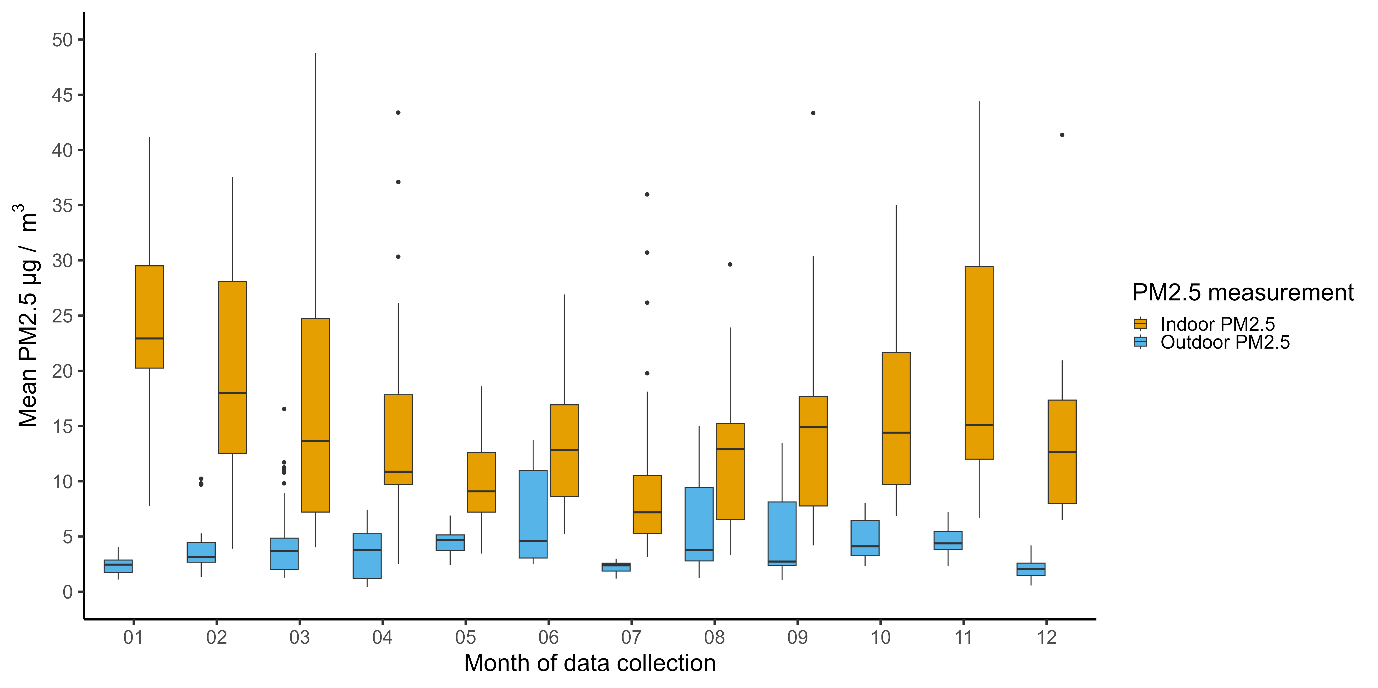
**

*Automatic Urban and Rural Network, Department for Environment, Food, & Rural Affairs (https://uk-air.defra.gov.uk/networks/network-info?view=aurn)

**Table S1. Descriptive information regarding dimensions of rooms with sensors and sensor placement relative to windows (309 homes).**

| **Room** | **Dimension** | **Mean size (metres)** | **SD size (metres)** | **Median size (metres)** | **Min size (metres)** | **Max size (metres)** |
| --- | --- | --- | --- | --- | --- | --- |
| Living/dining | Length | 4.87 | 1.29 | 4.48 | 2.41 | 10.27 |
|  | Width | 3.71 | 0.61 | 3.67 | 2.15 | 6.69 |
|  | Height | 2.49 | 0.25 | 2.42 | 2.01 | 3.37 |
| Kitchen | Length | 4.72 | 1.41 | 4.47 | 2.11 | 9.36 |
|  | Width | 3.18 | 0.91 | 3.02 | 1.43 | 6.69 |
|  | Height | 2.44 | 0.23 | 2.39 | 1.86 | 3.40 |
| Bedroom | Length | 3.61 | 0.96 | 3.51 | 0.33 | 8.92 |
|  | Width | 2.92 | 0.69 | 2.85 | 1.17 | 6.05 |
|  | Height | 2.40 | 0.32 | 2.38 | 0.54 | 5.35 |

**Table S2. Descriptive information regarding sensor placement relative to windows (309 homes).**

|  | **No window in room (*n* homes)** | **Cardinal directions window is facing (*n* windows)** | | | | | | | | **Are windows in room covered (blinds/ curtains/shutters; *n* windows)** | | | | |
| --- | --- | --- | --- | --- | --- | --- | --- | --- | --- | --- | --- | --- | --- | --- |
| **Room** |  | **N** | **NW** | **NE** | **S** | **SW** | **SE** | **W** | **E** | **None** | **Some** | **All** | **N/A** |  |
| Living/dining | 10 | 46 | 42 | 30 | 39 | 43 | 52 | 51 | 39 | 9 | 13 | 276 | 11 |  |
| Kitchen | 7 | 46 | 63 | 41 | 35 | 39 | 35 | 56 | 31 | 68 | 26 | 209 | 6 |  |
| Bedroom | 8 | 42 | 46 | 36 | 28 | 33 | 41 | 59 | 35 | 17 | 4 | 281 | 7 |  |

The mean number of windows per home was 12 (SD = 5); the minimum was 4 and the maximum was 36. This information was not collected at the room level.

**Table S3. Mean (*M*) and standard deviation (*SD*) of total indoor PM_2.5_ concentration using PM_2.5_ 24-hour metrics by room across the two week data collection period.**

| **Variable** | **Levels (*n*, % of sensor sample)** | **Daily average indoor PM_2.5_ (μg/m^3^)** | | | | | | | | | **Monitored hours, hourly average indoor PM_2.5_ concentration > 100μg/m^3^ (%)** | | | | | | **Monitored days, daily average PM_2.5_ concentration > 15μg/m^3^ (%)** | | | | | | | |
| --- | --- | --- | --- | --- | --- | --- | --- | --- | --- | --- | --- | --- | --- | --- | --- | --- | --- | --- | --- | --- | --- | --- | --- | --- |
|  |  | ***Kitchen*** | | | ***Living room*** | | | ***Child’s bedroom*** | | | ***Kitchen*** | | ***Living room*** | | ***Child’s bedroom*** | | ***Kitchen*** | | | ***Living room*** | | | ***Child’s bedroom*** | |
|  |  | ***M*** | ***SD*** | ***M*** | | ***SD*** | ***M*** | | ***SD*** | ***M*** | | ***SD*** | ***M*** | ***SD*** | ***M*** | ***SD*** | ***M*** | ***SD*** | ***M*** | | ***SD*** | ***M*** | | ***SD*** |
| Overall | *N = 309*, 100% | 23.5 | 34.6 | 19.7 | | 28.3 | 17.4 | | 22.4 | 5 | | 9 | 4 | 9 | 3 | 6 | 45 | 32 | 38 | | 32 | 35 | | 31 |
| Ethnicity | Other (*32*, 10%) | 17.2 | 20.5 | 18.4 | | 27.7 | 14.4 | | 17.9 | 3 | | 6 | 4 | 9 | 2 | 6 | 34 | 30 | 31 | | 30 | 27 | | 27 |
|  | South Asian (*150*, 49%) | 29.1 | 39.9 | 22.6 | | 27.8 | 18.9 | | 21.3 | 7 | | 9 | 5 | 8 | 3 | 5 | 57 | 30 | 48 | | 32 | 42 | | 31 |
|  | White (*127*, 41%) | 18.5 | 29.1 | 16.6 | | 28.7 | 16.4 | | 24.4 | 4 | | 10 | 3 | 10 | 3 | 7 | 32 | 28 | 28 | | 28 | 28 | | 31 |
| Housing tenure | Own (*236*, 76%) | 23.0 | 35.9 | 18.8 | | 28.3 | 15.3 | | 19.5 | 5 | | 9 | 4 | 9 | 2 | 5 | 43 | 32 | 36 | | 31 | 31 | | 30 |
|  | Rent (*73*, 24%) | 25.4 | 30.0 | 22.7 | | 28.1 | 24.0 | | 28.8 | 6 | | 9 | 5 | 8 | 5 | 8 | 49 | 31 | 45 | | 32 | 46 | | 34 |
| IMD-2019 BFD quintile | 1^st^ quintile (most deprived, *65*, 21%) | 26.6 | 28.6 | 22.7 | | 25.3 | 22.4 | | 25.6 | 6 | | 7 | 5 | 6 | 5 | 6 | 55 | 32 | 49 | | 33 | 45 | | 34 |
|  | 2^nd^ quintile (*84*, 27%) | 26.1 | 28.4 | 22.4 | | 27.4 | 19.3 | | 22.6 | 6 | | 9 | 5 | 9 | 4 | 7 | 53 | 31 | 45 | | 32 | 40 | | 32 |
|  | 3^rd^ quintile (*82*, 27%) | 25.4 | 50.2 | 20.8 | | 36.7 | 15.9 | | 22.6 | 6 | | 14 | 5 | 13 | 3 | 6 | 39 | 32 | 35 | | 31 | 31 | | 31 |
|  | 4^th^ quintile (*49*, 16%) | 16.9 | 23.2 | 13.5 | | 20.6 | 12.3 | | 17.7 | 3 | | 6 | 2 | 4 | 2 | 4 | 32 | 29 | 24 | | 26 | 23 | | 25 |
|  | 5^th^ quintile (least deprived, *23*, 7%) | 13.8 | 15.2 | 11.8 | | 14.4 | 12.9 | | 18.1 | 2 | | 3 | 1 | 3 | 2 | 4 | 30 | 28 | 23 | | 25 | 24 | | 26 |
|  | Missing (*6*, 2%) | 18.4 | 14.4 | 14.7 | | 11.8 | 15.8 | | 13.5 | 3 | | 3 | 1 | 2 | 1 | 2 | 48 | 20 | 36 | | 22 | 36 | | 19 |
| Child asthma status | Asthma (*144*, 47%) | 21.3 | 24.8 | 18.0 | | 22.5 | 16.4 | | 21.4 | 4 | | 6 | 3 | 6 | 3 | 6 | 44 | 31 | 37 | | 32 | 33 | | 32 |
|  | No asthma (*164*, 53%) | 25.5 | 41.2 | 21.3 | | 32.6 | 18.2 | | 23.3 | 6 | | 11 | 5 | 11 | 3 | 6 | 45 | 33 | 39 | | 32 | 37 | | 31 |
|  | Missing (*1,* <1%) | - | - | - | | - | - | | - | - | | - | - | - | - | - | - | - | - | | - | - | | - |
| Smoking household | Smoker (*117*, 38%) | 31.6 | 47.9 | 26.9 | | 38.1 | 22.5 | | 27.3 | 8 | | 14 | 6 | 13 | 5 | 8 | 53 | 34 | 47 | | 35 | 43 | | 35 |
|  | Non-smoker (*188*, 61%) | 18.5 | 21.6 | 15.3 | | 18.9 | 14.1 | | 17.9 | 4 | | 5 | 3 | 4 | 2 | 3 | 39 | 30 | 32 | | 28 | 29 | | 28 |
|  | Missing (*4*, 1%) | 22.1 | 18.3 | 15.2 | | 16.0 | 16.9 | | 19.2 | 4 | | 3 | 1 | 1 | 2 | 1 | 53 | 30 | 38 | | 16 | 36 | | 19 |
| Pets | No pets (*174*, 56%) | 23.4 | 29.1 | 20.3 | | 28.9 | 16.8 | | 21.2 | 5 | | 8 | 4 | 9 | 3 | 6 | 47 | 32 | 40 | | 32 | 35 | | 31 |
|  | Has pets (*135*, 44%) | 23.7 | 40.5 | 19.0 | | 27.5 | 18.1 | | 23.8 | 5 | | 11 | 4 | 9 | 3 | 6 | 41 | 32 | 35 | | 32 | 35 | | 32 |
| Age of building | Pre-1914 (*56*, 18%) | 18.4 | 22.7 | 16.7 | | 22.9 | 16.8 | | 22.6 | 3 | | 5 | 3 | 5 | 3 | 5 | 40 | 29 | 36 | | 29 | 36 | | 28 |
|  | Between 1914 – 1964 (*67*, 22%) | 27.5 | 52.1 | 22.0 | | 34.5 | 20.4 | | 27.3 | 6 | | 14 | 5 | 12 | 4 | 8 | 43 | 32 | 38 | | 32 | 38 | | 33 |
|  | Between 1965 – 1990 (*33*, 11%) | 28.2 | 41.2 | 26.9 | | 44.2 | 21.2 | | 26.7 | 7 | | 14 | 6 | 16 | 4 | 10 | 48 | 35 | 43 | | 34 | 39 | | 31 |
|  | After 1991 (*50*, 16%) | 22.2 | 28.0 | 16.6 | | 22.3 | 14.0 | | 19.3 | 5 | | 7 | 3 | 5 | 2 | 4 | 42 | 31 | 31 | | 30 | 27 | | 30 |
|  | Missing (*103*, 33%) | 23.0 | 24.5 | 19.2 | | 21.2 | 16.1 | | 17.7 | 5 | | 7 | 4 | 5 | 2 | 4 | 49 | 33 | 41 | | 33 | 35 | | 32 |
| Type of building | Detached/bungalow (*48*, 16%) | 18.6 | 24.2 | 14.0 | | 18.3 | 12.8 | | 16.0 | 4 | | 7 | 2 | 3 | 1 | 2 | 36 | 28 | 28 | | 26 | 26 | | 22 |
|  | Flat (*5*, 2%) | 18.9 | 14.6 | 18.8 | | 14.5 | 18.3 | | 15.4 | 3 | | 4 | 4 | 4 | 4 | 4 | 52 | 37 | 51 | | 35 | 52 | | 37 |
|  | Semi-detached (*150*, 49%) | 23.7 | 39.3 | 20.5 | | 30.1 | 18.9 | | 25.3 | 5 | | 10 | 4 | 10 | 4 | 7 | 44 | 32 | 38 | | 32 | 36 | | 32 |
|  | Terraced (*106*, 34%) | 25.8 | 31.7 | 21.3 | | 29.5 | 17.3 | | 20.5 | 6 | | 10 | 4 | 9 | 3 | 6 | 49 | 33 | 41 | | 33 | 36 | | 33 |
| Cooking appliance | Electric (*107*, 35%) | 19.3 | 26.6 | 16.9 | | 26.1 | 17.0 | | 25.8 | 4 | | 7 | 3 | 7 | 3 | 7 | 37 | 30 | 31 | | 29 | 30 | | 30 |
|  | Gas (*198*, 64%) | 26.1 | 38.3 | 21.4 | | 29.5 | 17.8 | | 20.5 | 6 | | 10 | 5 | 10 | 3 | 6 | 49 | 32 | 42 | | 33 | 38 | | 32 |
|  | Missing (*4*, 1%) | 11.6 | 11.9 | 9.3 | | 8.1 | 7.0 | | 6.9 | 2 | | 2 | 1 | 1 | 0 | 0 | 23 | 20 | 14 | | 10 | 16 | | 21 |
| Season of sensor deployment | Winter (*67*, 22%) | 29.8 | 28.2 | 24.1 | | 25.7 | 21.1 | | 22.3 | 7 | | 7 | 5 | 7 | 4 | 5 | 64 | 28 | 53 | | 33 | 47 | | 32 |
|  | Spring (*93*, 30%) | 25.6 | 48.5 | 20.4 | | 34.6 | 17.0 | | 22.7 | 5 | | 13 | 4 | 12 | 3 | 7 | 41 | 31 | 33 | | 30 | 31 | | 30 |
|  | Summer (*77*, 25%) | 15.2 | 19.9 | 14.5 | | 21.9 | 13.1 | | 17.9 | 3 | | 5 | 2 | 7 | 2 | 5 | 31 | 28 | 29 | | 27 | 25 | | 27 |
|  | Autumn (*72*, 23%) | 23.6 | 27.6 | 20.2 | | 26.4 | 18.9 | | 25.3 | 5 | | 7 | 4 | 7 | 4 | 7 | 46 | 31 | 40 | | 32 | 39 | | 33 |

**Table S4. Descriptive statistics showing spread of data for mean indoor PM_2.5_ concentration values from AirGradient sensors within homes and outdoor background PM_2.5_ concentration values from Bradford Council and AURN (DEFRA)* data by month, corresponding to Figure S5, collapsing all years.**

| **Month collected** | **N homes** | **Indoor PM_2.5_ (μg/m^3^)** | | | | | **Outdoor PM_2.5_ (μg/m^3^)** | | | | |
| --- | --- | --- | --- | --- | --- | --- | --- | --- | --- | --- | --- |
|  |  | **Mean** | **SD** | **Median** | **Min** | **Max** | **Mean** | **SD** | **Median** | **Min** | **Max** |
| January (01) | 21 | 23.2 | 9.3 | 22.9 | 7.7 | 41.2 | 2.3 | 0.8 | 2.5 | 1.1 | 4.0 |
| February (02) | 18 | 19.6 | 9.4 | 18.0 | 3.9 | 37.6 | 4.2 | 2.8 | 3.1 | 1.3 | 10.2 |
| March (03) | 33 | 16.9 | 12.1 | 13.6 | 4.0 | 48.8 | 4.8 | 3.9 | 3.7 | 1.2 | 16.5 |
| April (04) | 29 | 15.7 | 10.1 | 10.9 | 2.5 | 43.4 | 3.5 | 2.3 | 3.8 | 0.4 | 7.4 |
| May (05) | 25 | 10.2 | 4.1 | 9.1 | 3.5 | 18.6 | 4.5 | 1.1 | 4.7 | 2.4 | 6.9 |
| June (06) | 25 | 13.2 | 6.2 | 12.8 | 5.2 | 26.9 | 6.7 | 4.2 | 4.6 | 2.5 | 13.7 |
| July (07) | 22 | 11.0 | 9.2 | 7.2 | 3.1 | 36.0 | 2.2 | 0.5 | 2.4 | 1.2 | 3.0 |
| August (08) | 27 | 12.4 | 6.6 | 12.9 | 3.3 | 29.6 | 6.0 | 4.4 | 3.8 | 1.3 | 15.0 |
| September (09) | 24 | 14.9 | 8.9 | 14.9 | 4.2 | 43.3 | 5.3 | 4.3 | 2.7 | 1.1 | 13.4 |
| October (10) | 23 | 16.9 | 8.8 | 14.4 | 6.9 | 35.0 | 4.7 | 1.8 | 4.1 | 2.3 | 8.0 |
| November (11) | 17 | 20.6 | 11.5 | 15.1 | 6.7 | 44.4 | 4.6 | 1.5 | 4.4 | 2.3 | 7.2 |
| December (12) | 18 | 14.1 | 8.3 | 12.7 | 6.5 | 41.4 | 2.0 | 0.9 | 2.0 | 0.6 | 4.2 |

*Automatic Urban and Rural Network, Department for Environment, Food, & Rural Affairs (https://uk-air.defra.gov.uk/networks/network-info?view=aurn)

**Table S5. Total indoor PM_2.5_ levels by different types of heating available in home (does not account for use).**

| **Levels** | ***N*** | **Daily average indoor PM_2.5_ (μg/m^3^)** | | **Monitored hours, hourly average indoor PM_2.5_ exposure > 100μg/m^3^ (%)** | | **Monitored days, daily average indoor PM_2.5_ exposure > 15μg/m^3^ (%)** | |
| --- | --- | --- | --- | --- | --- | --- | --- |
|  |  | ***M*** | ***SD*** | ***M*** | ***SD*** | ***M*** | ***SD*** |
| Gas central only | 198 | 19.3 | 25.9 | 4 | 7 | 39 | 31 |
| Gas central and portable heaters: electric, bottle gas/paraffin, oil-filled | 37 | 20.8 | 23.1 | 4 | 6 | 46 | 34 |
| Gas central and fixed gas fire | 32 | 21.4 | 23.2 | 5 | 8 | 46 | 36 |
| Gas central and fixed electric | 16 | 26.2 | 32.9 | 6 | 9 | 48 | 32 |
| Gas central and wood stove (solid fuel/wood/coal) | 16 | 19.4 | 20.3 | 4 | 7 | 42 | 28 |
| Gas central and open fires | 2 | 6.5 | 3.6 | 0 | 0 | 3 | 5 |
| Gas fixed fire only* | 2 | 60.9 | 44.5 | 21 | 24 | 86 | 20 |
| Fixed electric only | 2 | 13.7 | 11.3 | 2 | 1 | 27 | 18 |
| Portable heaters: electric, bottle gas/paraffin, oil-filled only | 1 | 19.6 | 8.9 | 1 | - | 69 | - |
| Fixed electric and fixed gas fire | 1 | 10.4 | 5.5 | 0 | - | 12 | - |
| Open fires and portable heaters: electric, bottle gas/paraffin, oil-filled | 1 | 3.5 | 2.1 | 0 | - | 0 | - |
| Gas central and oil central heating | 1 | 47.7 | 24.8 | 11 | - | 100 | - |

*These high levels were driven by one home
